# Modular Metabolic Engineering and Synthetic Coculture Strategies for the Production of Aromatic Compounds in Yeast

**DOI:** 10.1021/acssynbio.3c00047

**Published:** 2023-05-23

**Authors:** Huadong Peng, Ruiqi Chen, William M. Shaw, Piotr Hapeta, Wei Jiang, David J. Bell, Tom Ellis, Rodrigo Ledesma-Amaro

**Affiliations:** † Department of Bioengineering, Imperial College London, London SW7 2AZ, U.K.; ‡ Centre for Synthetic Biology, Imperial College London, London SW7 2AZ, U.K.; § College of Life Sciences, Nankai University, Tianjin 300071, China; ∥ SynbiCITE Innovation and Knowledge Centre, Imperial College London, London SW7 2AZ, U.K.

**Keywords:** synthetic biology, combinatorial engineering, division of labor, microbial communities, *p*-coumaric acid, raspberry ketone

## Abstract

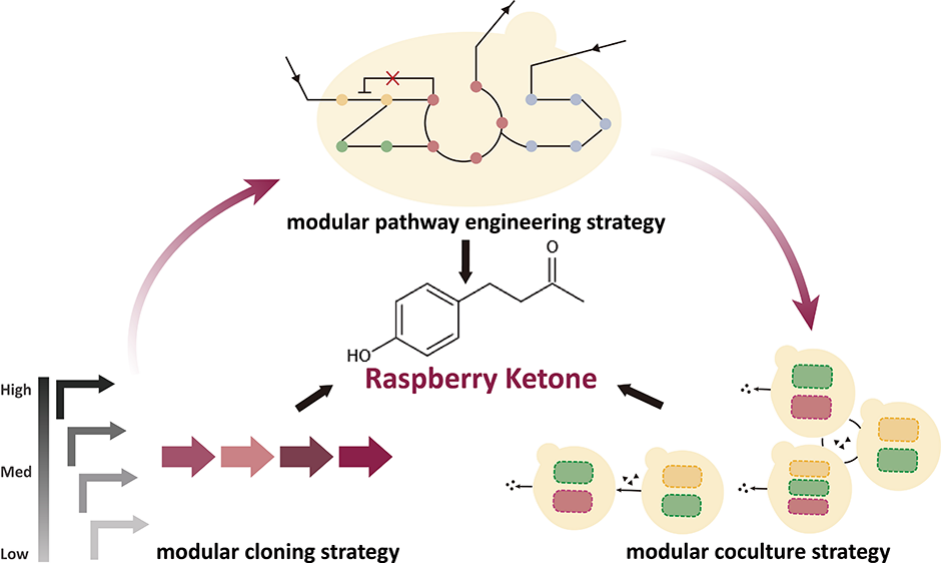

Microbial-derived aromatics provide a sustainable and renewable alternative to petroleum-derived chemicals. In this study, we used the model yeast *Saccharomyces cerevisiae* to produce aromatic molecules by exploiting the concept of modularity in synthetic biology. Three different modular approaches were investigated for the production of the valuable fragrance raspberry ketone (RK), found in raspberry fruits and mostly produced from petrochemicals. The first strategy used was modular cloning, which enabled the generation of combinatorial libraries of promoters to optimize the expression level of the genes involved in the synthesis pathway of RK. The second strategy was modular pathway engineering and involved the creation of four modules, one for product formation: RK synthesis module (Mod. RK); and three for precursor synthesis: aromatic amino acid synthesis module (Mod. Aro), *p*-coumaric acid synthesis module (Mod. *p*-CA), and malonyl-CoA synthesis module (Mod. M-CoA). The production of RK by combinations of the expression of these modules was studied, and the best engineered strain produced 63.5 mg/L RK from glucose, which is the highest production described in yeast, and 2.1 mg RK/g glucose, which is the highest yield reported in any organism without *p*-coumaric acid supplementation. The third strategy was the use of modular cocultures to explore the effects of division of labor on RK production. Two two-member communities and one three-member community were created, and their production capacity was highly dependent on the structure of the synthetic community, the inoculation ratio, and the culture media. In certain conditions, the cocultures outperformed their monoculture controls for RK production, although this was not the norm. Interestingly, the cocultures showed up to 7.5-fold increase and 308.4 mg/L of 4-hydroxy benzalacetone, the direct precursor of RK, which can be used for the semi-synthesis of RK. This study illustrates the utility of modularity in synthetic biology tools and their applications to the synthesis of products of industrial interest.

## Introduction

Aromatics have a wide range of applications in the food, pharmaceutical, and chemical industries. Raspberry ketone (RK, also known as 4-(*p*-hydroxyphenyl)-2-butanone) is an expensive ingredient in the flavor and fragrance industry. While it is naturally produced in low amount in raspberry fruits (1 g RK per 1000 kg of fruits), its current production is mainly from petroleum-derived chemicals, which is not sustainable.^1^ However, microbial-derived RK provides an alternative production method, which is environmentally friendly and renewable.

Over the past years, there have been a few studies aimed to test the potential of RK production in both *E. coli* and *S. cerevisiae* (Table 1)*.* The natural synthesis of RK initiates with the phenylpropanoid pathway in the fruit of raspberry (*Rubus idaeus*). *p*-Coumaric acid (*p*-CA) is first activated to 4-coumaroyl-CoA by 4-coumaroyl-CoA ligase (4CL), followed by condensation with one malonyl-CoA to 4-hydroxy benzalacetone (HBA) by benzalacetone synthase (BAS) and sequential reduction to RK via RK synthase (RKS).^1,2^ Most of the early studies for the heterologous production of RK supplemented the culture media with the precursor *p*-CA. Taking this approach, one of the first reports of microbially produced RK, combined the supplementation of 3 mM *p*-CA, with the co-expression of *4CL* and *RiCHS*, producing around 5 mg/L RK in *E. coli*, while no production was found in yeast, which only made the precursor HBA 0.06 mg/L.^1^ Another work expressed the RK synthesis pathway in *S. cerevisiae* by expressing *RtPAL*, *AtC4H*, *At4CL1*, *Pc4CL2*, and *RpBAS*. The engineered *S. cerevisiae* produced 2.8 mg/L RK from glucose and 7.5 mg/L RK with 3 mM *p*-CA supplementation.^3^ In another two examples, the three genes *4CL*, *BAS*, and *RiRZS1* were introduced into *E. coli*. In the first case, the copy numbers of these three genes were optimized to generate the best producer strain, which achieved 90.97 mg/L in TK medium supplemented with 300 mg/L *p*-CA.^4^ In the second study, the final engineered *E. coli* reached 203 mg/L RK in a 1 L fermenter using soybean oil and glycerol (1:1) as substrate, with a supplementation of *p*-CA at 2.5 mmol/L/12 h.^5^ While RK titer has been greatly improved in the recombinant strains, the need for *p*-CA supplementation is undesired for production at scale due to the cost and nonrenewable origin of the precursor. Hence, it is necessary to develop efficient de novo RK synthesis from available substrates such as glucose.

**Table 1:** Raspberry Ketone Synthesis and Production in Microbial Cell Factories

microbial host	genetic modifications	titer mg/L, yield mg/g glucose	medium/precursor feeding	plate, flask, bioreactor	references
With *p*-Coumaric Acid/Chemical Feeding					
*E. coli BL21*	*pAC-4CL-RiCHS*	no HBA,5 mg/L RK	2× YT, IPTG induction,3 mM *p*-CA	800 mL, fermenter	(1)
*E. coli* BL21	multiple copies of *At4CL1*, *RpBAS*, *RiRZS1*	∼5 mg/L HBA,90.97 mg/RK	TB medium (with 5 g/L glucose), 300 mg/L *p*-CA	N.A.	(4)
*E. coli* BL21	Δ*tyrR::Zeo^r^*, *tyrA*, *aroG^fbr^*, *RgPAL*, *AtCL*, *RpBAS^S331V^*, *fabF*	62 mg/L RK,0.71 mg RK/g glucose	fermentation medium, 0.1 mM IPTG, 100 nm cerulenin, glucose (0.8 g/L/h)	0.5 L jar fermenter	(20)
*E. coli* CR8	*4CL1*, *BAS*, *RZS1* based on fatty acid synthesis strain	203 mg/L RK	soybean oil:glycerol 1:1, *p*-CA 2.5 mmol/L/12 h,	1 L, bioreactor	(5)
*E. coli*	*RiRZS1*, *SyGDH*	9.89 g/L RK	25 g/L glucose, 10 g/L HBA	5 mL, flask	(21)
*Corynebacterium glutamicum*	*M-CoA ΔldhA*, *RpBAS*, *EccurA*, *EcudhA*	99.8 mg/L, 2.50 mg RK/g glucose	defined CGXII medium with 4% (w/v) glucose, 1 mM IPTG, fed *p*-CA, HBA	50 mL, flask	(22)
*S. cerevisiae* YPH499	pESC-*4CL-RiCHS*	0.06 mg/L HBA,no RK	YPGal-medium, galactose induction, 3 mM *p*-CA	50 mL, flask	(1)
*S. cerevisiae*	*RtPAL*, *AtC4H*, *At4CL1*, *Pc4CL2*, *RpBAS*	7.5 mg/L RK, 0.38 mg RK/g glucose	YPD, 3 mM *p*-CA	100 mL, flask	(3)
Without *p*-Coumaric Acid/Chemical Feeding					
*E. coli* DH10β	*TAL*, *PCL*, *BAS*, *RKS*, *MatB*	12.9 mg/L RK	2YT	microtiter plate	(12)
*E. coli* BL21	Δ*tyrR::Zeo^r^*, *tyrA*, *aroG^fbr^*, *RgPAL*, *AtCL*, *RpBAS^S331V^*, *fabF*	41 mg/L RK,0.47 mg RK/g glucose	fermentation medium, 0.1 mM IPTG, glucose (0.8 g/L/h)	0.5 L jar fermenter	(20)
*S. cerevisiae*	*RtPAL*, *AtC4H*, *At4CL1*, *Pc4CL2*, *RpBAS*	2.8 mg/L RK,0.14 mg RK/g glucose	YPD	100 mL, flask	(3)
*S. cerevisiae*	*RtTAL*, *At4CL*, *RpBAS*, *RiRKS*, *ScARO3^K222L^*, *ScARO4^K229L^*, *ScARO7^G141S^*, *VvPAL*, *AtC4H*, *FjTAL*, *ScALD6*, *SeACS1^L641p^*, *ScACC1*^*S659A*, *S1157A*^	158.8 mg/L HBA,17.3 mg/L RK, 0.87 mg RK/g glucose	SM, synthetic minimal medium	500 μL,96 well deep plate	this study
*S. cerevisiae*	same as above	**no HBA, 63.5 mg/L RK,2.1 mg RK/g glucose**	1.5× SM, synthetic minimal medium	25 mL, flask	this study
*S. cerevisiae*	coculture CL_RK1	308.4 mg/L HBA,6.8 mg/L RK, 0.34 mg RK/g glucose	SM, synthetic minimal medium	500 μL,96 well deep plate	this study
*S. cerevisiae*	coculture CL_RK3	280 mg/L HBA,13.3 mg/L RK, 0.67 mg RK/g glucose	SM, synthetic minimal medium	500 μL,96 well deep plate	this study

In the past couple of decades, synthetic biology has developed molecular techniques that allow us to engineer microorganisms in a more efficient manner. One of the concepts that synthetic biology has heavily promoted is modularity. By breaking down the complexity of biology into defined modules, we can better understand and engineer biological processes. Modular tools are especially useful because of their versatility and capacity to explore larger design spaces. This can be used for metabolic engineering, and some examples of these are (1) the use of modular cloning to generate libraries of expression cassettes, often focusing on promoter-ORF optimisation,^6−8^ (2) the use of modular pathway strategies, where complex metabolic pathways are split in smaller modules (synthesis of precursors, reduction of competing pathways, etc.),^9^ and (3) the use of modular cocultures or synthetic microbial communities where each member can be specialized in a specific task.^10,11^

Out of these three modular strategies, only modular cloning and combinatorial promoter engineering have been applied to RK production and only in *E. coli.*(12) Moore et al. used the toolkit EcoFlex^6^ to establish a combinatorial promoter library to optimize the expression levels of the genes in the RK synthesis pathway including *TAL*, *PCL*, *BAS*, *RKS*, and *MatB*. The final engineered *E. coli* produced 12.9 mg/L RK by complete synthesis from glucose.^12^ Modular pathway engineering, aimed at dividing the metabolism in rationally defined modules that can be engineered separately or combined, have been widely used to improve production levels.^9,13,14^ Modular pathway engineering has been used successfully for the production of other aromatic compounds, such as *p*-coumaric acid,^14^ tryptophan,^8^ and resveratrol.^13^ For the synthesis of RK, the pathways can be divided in modules, each leading to the synthesis of different precursors, aromatic acids, *p*-CA, and malonyl-CoA.

Moreover, modular coculture engineering via division of labor could be an effective approach to further improve bioproduction. Complex metabolic pathways often require excessive gene modifications, leading to metabolic burden, which may sacrifice cellular fitness or product titers.^10,11^ To address these issues, division of labor could be considered to split metabolic pathways within cocultures. Based on different inoculation ratios and assembly options, each member could be easily assembled with short pathways to fine-tune the metabolic fluxes and maximize product titers. There are some recent examples using modular cocultures for the production of aromatic compounds such as resveratrol,^15,16^ genistein,^17^ kaempferide,^18^ and caffeic acid^19^ but not yet for RK. In addition, most cases were tested in *E. coli*–*E. coli* cocultures. Thus, it would be interesting to apply yeast–yeast modular cocultures for RK production.

In this work, we have studied three different modular synthetic biology approaches to improve the production of RK in yeast. First, we used modular cloning to create a combinatorial promoter library to identify and optimize the module of RK synthesis pathway. Then, we adopted a modular pathway engineering strategy to further improve RK titer by including three additional modules to increase the availability of the precursors *p*-coumaric acid and malonyl-CoA. The best engineered strain produced 63.5 mg/L RK, which is the highest reported RK titer from glucose in *S. cerevisiae*, amounting to a yield of 2.1 mg RK/g glucose, which is the highest reported in any organism without *p*-coumaric acid supplementation. Finally, a modular coculture strategy was adopted to further explore RK production potential by creating two two-member cocultures and one three-member coculture. These cocultures demonstrated advantages in improving production only in certain conditions. The cocultures also produced increased amounts of the precursor HBA (308.4 mg/L), used in industry to synthesize RK. This study successfully demonstrated the potential of modular strategies, at different levels, to improve the microbial production of aromatic compounds such as RK.

## Results and Discussion

### Modular Cloning Strategy: Optimizing the RK Synthesis Pathway in Yeast Using Combinatorial Promoter Engineering

To begin our study of modular synthetic biology strategies for producing aromatic chemicals, we first looked to establish and optimize the production of raspberry ketone in yeast. It has previously been shown that yields of up to 2.8 mg/L of RK can be achieved in yeast when using synthetic fusions of the 4-coumarate-CoA ligase from *Arabidopsis thaliana* (At4CL) and benzalacetone synthase from *Rheum palmatum* (RpBAS) proteins.^3^ However, a recent study concluded that precise expression of the enzymes in this engineered pathway is paramount, and anything more than low expression of 4CL can lead to overall reduced yields of RK due the toxicity of intermediates, whereas higher expression of BAS is preferred.^12^ In this instance, combinatorial promoter libraries were used in *E. coli* to understand and tune the optimal expression of the individual pathway enzymes.

**Figure 1:**
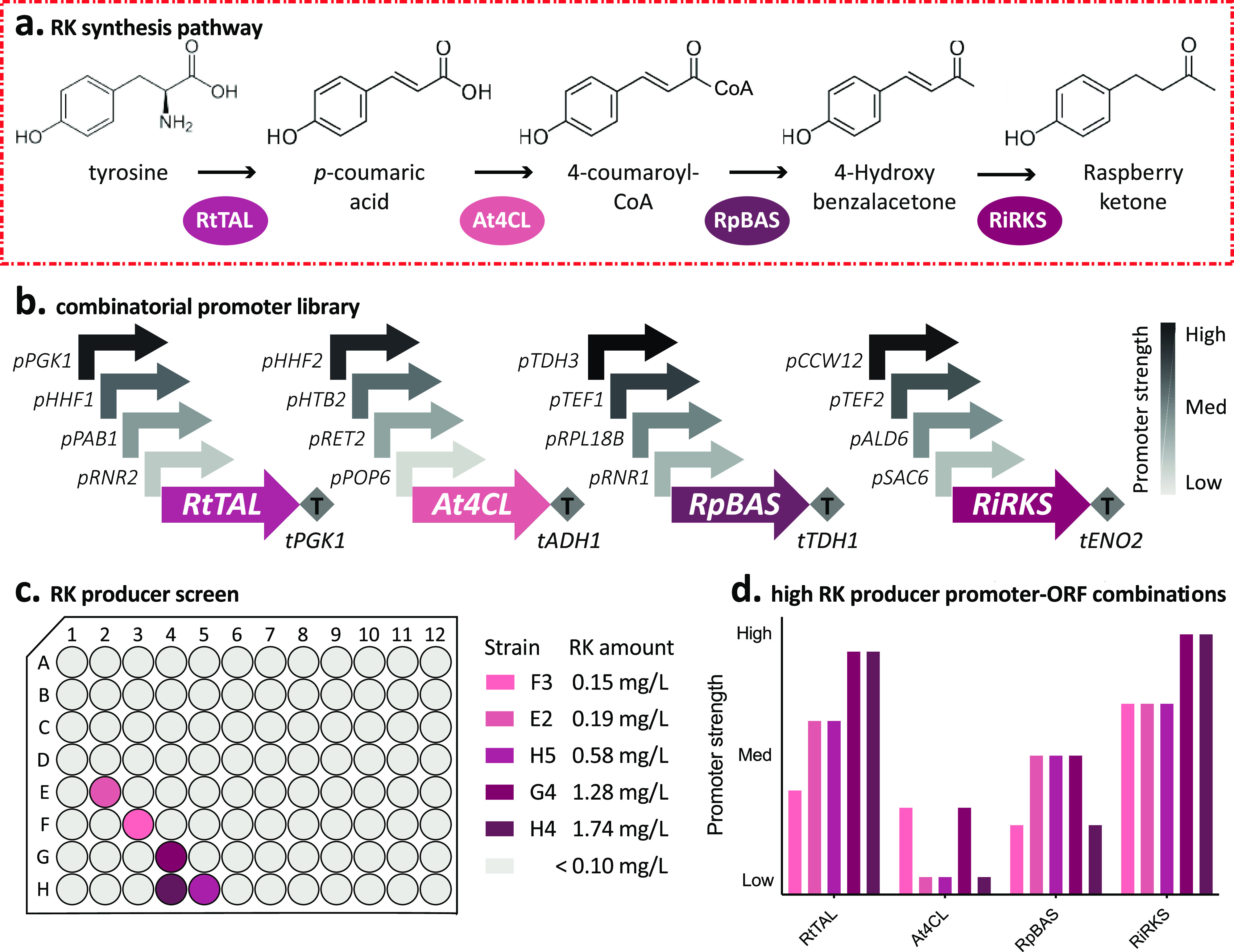
Raspberry ketone synthesis pathway refactoring in *Saccharomyces cerevisiae*. (a) RK synthesis pathway in *S. cerevisiae*, which begins with the conversion of L-tyrosine to *p*-coumaric acid by tyrosine ammonia lyase (TAL). Conversion of *p*-coumaric acid is then converted into RK in three additional enzymatic steps, by *p*-coumaroyl-CoA synthetase (4CL), benzylacetone synthase (BAS), and raspberry ketone reductase (RKS). (b) Promoter-ORF combinations used in the combinatorial promoter library for tuning the expression of the RK synthesis pathway enzymes. (c) RK titers of 96 randomly selected strains after 3 days of growth in SC minus uracil media. Experimental measurements are RK amounts as determined by LC–MS from spent media and shown as individual values from a single replicate. (d) Relative promoter strengths of the RK synthesis pathway genes from the five strains identified in the RK producer screen. Promoter strengths are rank order (1–15) corresponding to their relative promoter strength as characterized in the Yeast MoClo Toolkit.^7^

To explore whether a modular combinatorial promoter engineering approach could also lead to increased RK production in yeast, we introduced four enzymes from the raspberry ketone synthesis pathway: tyrosine ammonia-lyase (*RtTAL*), 4-coumarate-CoA ligase (*At4CL*), benzalacetone synthase (*RpBAS*), and raspberry ketone synthase (*RiRKS*) (Figure 1a). We amplified the open reading frames (ORFs) of the four genes described by Moore et al.^12^ from the *E. coli* plasmids and cloned them into the Yeast MoClo Toolkit (YTK) ecosystem.^7^ To determine whether the genes expressed in yeast, we created C-terminal fusions of the four proteins with GFP and individually assessed relative expression using flow cytometry (Figure S1a). RtTAL, At4CL, and RiRKS expressed well in yeast. However, no expression was seen for RpBAS, and so we codon-optimized this ORF for *S. cerevisiae*, which demonstrated greatly improved expression (Figure S1b).

With all four RK synthesis pathway enzymes expressing well in yeast, we next set out to understand the optimal expression levels that would produce the greatest yields of RK. We created a combinatorial promoter library, expressing each ORF from four different promoters, ranging from strong to weak, as characterized in the YTK^7^ (Figure 1b). We then randomly assembled all promoter-ORF combinations in a one-pot Golden Gate reaction into a high-copy 2 μ plasmid, with a *URA3* selection marker, so that each ORF would be represented once with any one of the four chosen promoters. The randomized plasmid assembly was then prepared as library containing >10,000 isolates and transformed in to wild-type BY4741 yeast. Ninety-six colonies were randomly chosen and grown in 500 μL of synthetic complete (SC) minus uracil media in a deep-well plate for 3 days at 30 °C and 700 rpm (Figure 1c). We then spun down the cultures and sampled the supernatant for direct measurement of RK by LC–MS. From the 96 randomly selected strains, five produced a measurable amount of RK above 0.1 mg/L, with the top strain producing 1.7 mg/L RK. It is worth noting that screening a higher number of isolates could lead to higher producer strains, and it is something to consider in future optimization studies.

To identify the promoter-ORF combinations that led to the highest levels of RK, we isolated the 2 μ plasmids from the five yeast strains and sequenced the promoter regions of the four genes. This revealed a trend toward low expression of 4CL, as seen in a previous work, modest expression of BAS, and high expression of RtTAL and RiRKS (Figure 1d). These results agree with previous work in *E. coli* that showed that low levels of 4CL were critical, highlighting the importance of identifying the optimal expression levels of each protein in pathways that can produce toxic intermediates.^12^ Although 1.7 mg/L RK is not as high the 2.8 mg/L achieved by Lee et al.^3^ using phenylalanine pathway and synthetic fusion protein of 4CL and BAS in yeast, this was a reasonable yield without additional growth optimization and a good starting place to explore additional modular strategies for increasing production further. As strain H4 demonstrated the highest titers of RK, we chose the *pPGK1-RtTAL*, *pPOP6-At4CL*, *pRNR1-RpBAS*, and *pCCW12-RiRKS* promoter-ORF combinations for the final RK synthesis module (Mod. RK), which would be stably integrated into the yeast genome.

### Modular Pathway Engineering Strategy: Optimizing RK Production by Metabolic Engineering

**Figure 2:**
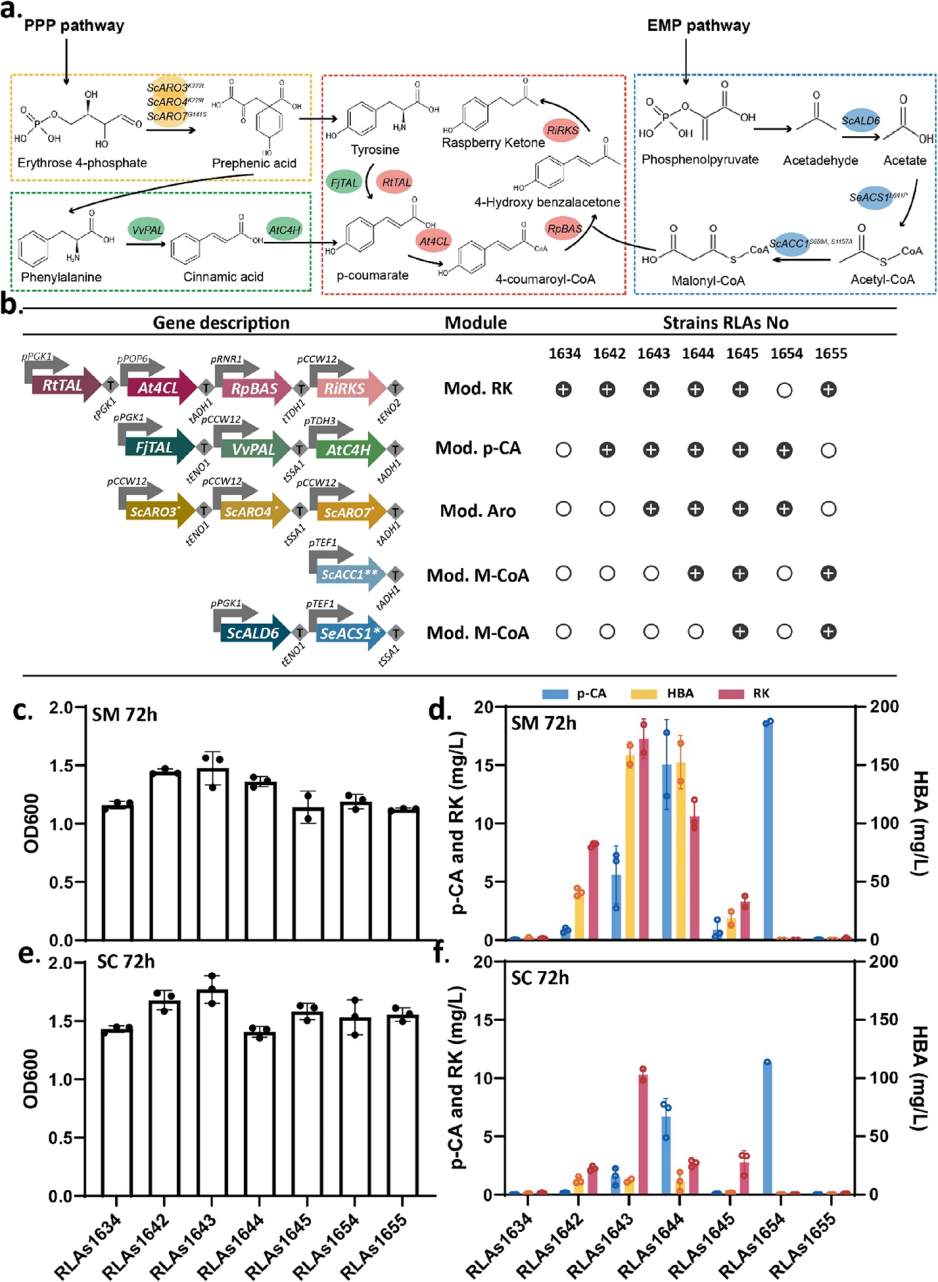
Modular metabolic engineering strategy to improve raspberry ketone synthesis and production in *S. cerevisiae*. (a) Four modules are introduced for the RK synthesis pathway, and the enzymes of each module are grouped by colors. Mod. RK (red) is described in Figure 1, Mod. Aro (yellow): overexpression of *ScARO3^K222L^*, *ScARO4^K229L^*: DAHP synthase; *ScARO7^G141S^*: chorismate mutase; Mod. *p*-CA (green): overexpression of *VvPAL*: phenylalanine ammonia-lyase from *Vitis vinifera; AtC4H*: cinnamate-4-hydroxylase from *Arabidopsis thaliana*; *FjTAL*: tyrosine ammonia-lyase from *Flavobacterium johnsoniae;* Mod. M-CoA (blue): overexpression of *ScALD6*: aldehyde dehydrogenase from *S. cerevisiae*; *SeACS1^L641p^*: acetyl-CoA synthetase with mutate site L641P from *Salmonella enterica*; *ScACC1*^*S659A*,*S1157A*^: acetyl-CoA carboxylase with two mutation sites: S659A, S1157A from *S. cerevisiae.* (b) Description of gene combinations of each module and modular engineered strains. (c–f) Engineering strains are cultured at 30 °C and 250 rpm in 96 deep well plates in synthetic minimal medium (SM) and synthetic complete medium (SC) for 72 h. The OD values from plate reader are shown in (c) and (e). The precursors *p*-coumaric acid (*p*-CA), 4-hydroxy benzalacetone (HBA) and product RK are shown in (d) and (f). These results show the average of three replicates and the SD.

Having optimized the Mod. RK, we then adopted a modular metabolic engineering strategy to further optimize RK production in yeast (Figure 2). Alongside the Mod. RK**,** three other modules were designed to increase precursors supply toward RK production (Figure 2a). Mod. Aro was constructed to enhance aromatic amino acid synthesis by overexpressing the feedback inhibition version of DAHP synthase (*ScARO3^K222L^*, *ScARO4^K229L^*) and chorismate mutase (*ScARO7^G141S^*).^23−27^ Mod. *p*-CA was constructed to improve *p*-coumaric acid synthesis by the heterologous expression of phenylalanine ammonia-lyase from *Vitis vinifera* (*VvPAL*), cinnamate-4-hydroxylase from *Arabidopsis thaliana* (*AtC4H*), and tyrosine ammonia-lyase from *Flavobacterium johnsoniae* (*FjTAL*).^28−31^ Mod. M-CoA was designed to increase the acetyl-CoA and malonyl-CoA supply. Mod. M-CoA contained the expression of the aldehyde dehydrogenase *ScALD6*, a mutated acetyl-CoA synthetase (L641P) from *Salmonella entericaSeACS1^L641p^* and a mutated acetyl-CoA carboxylase (S659A, S1157A) *ScACC1*^*S659A*,*S1157A.*^(32,33) To increase modularity, Mod. M-CoA was split into two cassettes, one overexpressing *ScACC1*^*S659A*,*S1157A*^ (aimed to increase malonyl-CoA production) and other overexpressing *ScALD6 and SeACS1^L641p^* (aimed to maximize acetyl-CoA synthesis, the direct precursor of malonyl-CoA).

First, we generated the strain RLAs1634, which expressed the optimized Mod. RK in the LEU2 locus. To evaluate the effect of each precursor module, Mod. *p*-CA (via the HO or TRP1 locus), Mod. Aro (via the URA3 or HO Locus), and Mod. M-CoA (via the HO Locus) were sequentially integrated in the strain RLAs1634 to form the engineered strains RLAs1642–45 and RLAs1654–55 (Figure 2b). These engineered strains were cultured in both SM and SC medium in 96-well deep plates at 30 °C and 250 rpm for 72 h.

The sequential addition of Mod. *p*-CA and Mod. Aro significantly improved RK titers and cell growth when compared to the parental strain, but it was not the case for the expression of Mod. M-CoA. As shown in Figure 2c,d in SM medium, strain RLAs1642 containing Mod. RK and Mod. *p*-CA achieved an RK titer of 8.2 mg/L, which was 74 times higher than that of the parental strain containing only Mod. RK (RLAs1634). Further addition of Mod. *p*-CA generated strain RLAs1643, which reached 17.3 mg/L RK, 2.1 times that of RLAs1642 and 157 times that of RLAs1634. Meanwhile, the addition of Mod. *p*-CA and Mod. Aro also brought an increase in the intermediates *p*-CA and HBA. Strain RLAs1643 accumulated 5 mg/L *p*-CA and 158.8 mg/L HBA. However, when we tried to further increase the RK titer by adding Mod. M-CoA, we found a drop in the RK titer to 10.2 mg/L with *ScACC1*^*S659A*,*S1157A*^ expression (strain RLAs1644) and to 3.3 mg/L with the expression of the full Mod. M-CoA (*ScALD6*, *SeACS1^L641p^*, *ScACC1*^*S659A*,*S1157A*^) (strain RLAs1645). To evaluate the independent effect of Mod. M-CoA, we created the strain RLAs1655 expressing Mod. RK and Mod. M-CoA, which did not increase RK levels when compared with the parental strain RLAs1634. In addition, high concentrations of intermediates *p*-CA (15.1 mg/L) and HBA (152.6 mg/L) were accumulated in RLAs1644, while they substantially decreased for RLAs1645. In accordance with the RK trend, biomass formation was also increased with Mod. *p*-CA and Mod. Aro addition but slightly reduced with Mod. M-CoA (Figure 2c). Strain RLAs1654 was designed as a *p*-CA sender strain for the following coculture setup, and it produced 18.7 mg/L *p*-CA in SM.

When comparing growth and production in two different media, minimal media SM and richer media SC, we observed similar trends but higher titers of both RK and intermediates in SM (Figure 2e,f). For example, the best RK producer RLAs1643 reached 10.3 mg/L RK in SC and 17.3 mg/L RK in SM. Further research would need to be done to understand the difference of bioproduction titers between SM and SC, and potential explanation could come from the presence of specific amino acids that could negatively regulate some pathways or by variations in the ratio of nitrogen to carbon, which is known to globally affect metabolism. Due to the lower cost of SM compared to SC, these results would benefit the cost-efficiency of the process. In addition, when the best RK producer RLAs1643 was grown in flask for 72 h, it achieved 63.5 mg/L RK and 333.9 mg/L p-CA (Figure S2), which suggest that further improvements in titer can be obtained by optimizing culture conditions.

As a summary, our modular metabolic engineering strategy successfully improved the de novo synthesis of RK and achieved the highest reported RK titer of 63.5 mg/L in a 50 mL flask in the yeast, *S. cerevisiae*. This strain also produced high amounts of HBA in 96 DWP, with titers of 158.8 mg/L, which can be used as precursor to chemically synthetize RK.^34^ The observed accumulation of HBA indicates a bottleneck in the last step of the pathway, which indicates either a low activity of *RiRKS* or a limited availability of NADPH required for that enzymatic step.^1^ Thus, the future metabolic engineering challenge for RK synthesis should consider the balanced supply of cofactor NADPH and the screening of highly efficient RK synthases. The highest RK production and better growth were achieved by combining Mod. RK, Mod. Aro, and Mod. *p*-CA (RLAs1643). In agreement with previous reports,^4,13^ we also observed that Mod. M-CoA was detrimental to both RK production titers and cell growth. Therefore, we decided not to use Mod. M-CoA in the following experiments.

### Modular Coculture Strategy: Exploring Division of Labor to Produce RK

**Figure 3:**
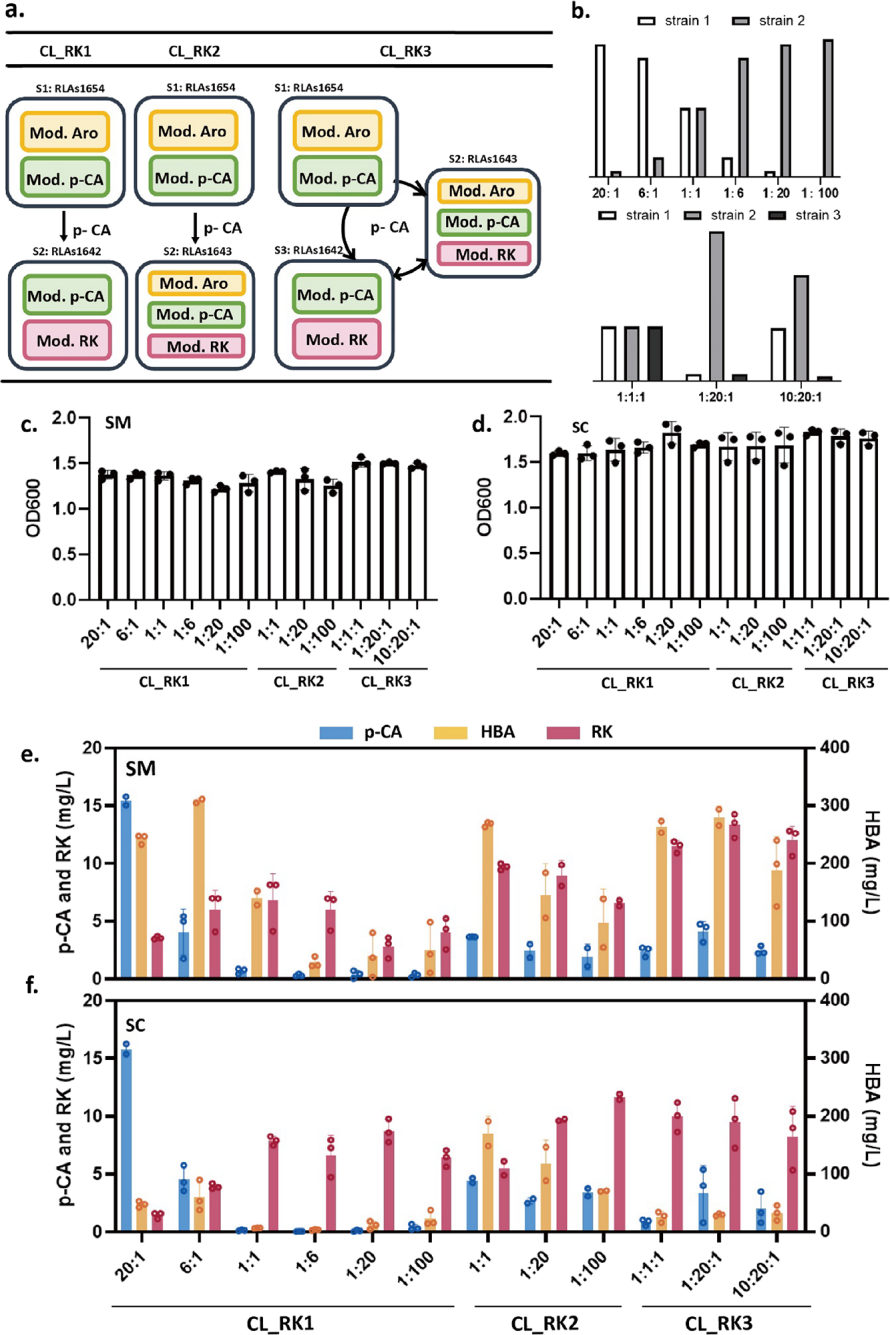
Modular coculture strategy via division of labor to explore the production of RK. (a) Diagram of modular coculture strategies: *p*-CA producing strain RLAs1654 was used as the sender strain (S1) and two receiver strains (S2, S3) including RLAs1642 and RLAs1643 based on their promising RK titers from monocultures. Two pairs of two-member cocultures (CL_RK1, CL_RK2) and one pair of three-member coculture (CL_RK3) were designed, and each member within these cocultures was communicated by *p*-CA diffusion. (b) Different initial inoculation ratios were selected to study the production of RK, *p*-CA, and HBA. The ratios for CL_RK1 were 20:1, 6:1, 1:1, 1:6, 1:20, and 1:100; for CL_RK2, they were 1:1, 1:20, and 1:100; and for CL_RK3, they were 1:1:1, 1:20:1, and 10:20:1. These three pairs of cocultures were cultured using 96-well deep plates in SM and SC for 72 h. (c, d) The OD values of cocultures in both SM and SC at 72 h from a microplate reader. (e, f) Titers of precursor *p*-CA and product HBA and RK at 72 h in both SM and SC. The values presented here are average of triplicates and SD.

Having explored modular cloning (promoter optimization) and modular pathway engineering, we decided to also explore the use of modular cells in RK production. Strains bearing different modifications can establish cocultures that, through division of labor, may improve bioproduction.^11^ To explore RK production in cocultures, three cocultures were designed (CL_RK1, CL_RK2, CL_RK3), all of them using the RLAs1654 strain as a *p*-CA producer and the RLAs1642 and/or RLAs1643 strains as RK producers (Figure 3a). They were based on the following observations: (1) *p*-CA, an important intermediate in RK synthesis, can diffuse in and out the yeast cells and (2) the heavily engineered strains RLAs1644 and RLAs1645 showed reduced cell growth potentially due to metabolic burden, which could be reduced by division of labor.

To test whether the initial inoculation ratio could be used to fine-tune the metabolic fluxes between *p*-CA and RK, different initial ratios were tested for the coculture CL_RK1 including 20:1, 6:1, 1:1, 1:6, 1:20, and 1:100. Coculture CL_RK1 showed an overall good cell growth in SM in different ratios, although its biomass formation is reduced with the increased proportion of the RK producer cells (RLAs1642), which grow slower (Figure 3c). The accumulation of *p*-CA followed the expected trend (Figure 3e), with a higher concentration of 15.4 mg/L *p*-CA at a 20:1 ratio, followed by 4.0 mg/L *p*-CA at a 6:1 ratio. Other ratios produced less than 0.7 mg/L *p*-CA. Interestingly, the precursor HBA showed relatively high concentrations, with a maximum titer of 308.4 mg/L at a 6:1 ratio, which was 7.5 times higher than that of RLAs1642 monoculture. At a 1:1 ratio, the maximum RK titer was achieved (8.2 mg/L), which equals that of the RLAs1642 monoculture, while the precursor HBA reached 140.3 mg/L, which was 3.4 times higher than that of the RLAs1642 monoculture. Other initial inoculation ratios reduced RK titers gradually. These results show that when varying inoculation ratios, different production profiles can be achieved, and metabolic fluxes can be optimized to produce specific products of interest.

As expected, the coculture CL_RK1 showed higher OD values in SC than in SM media (Figure 3d). The intermediate *p*-CA showed a similar performance in SC and SM, while the concentration of HBA was lower in SC (Figure 3f). The optimal ratio for RK production was also 1:1 in SC, but interestingly, high production titers were maintained (with a maximum at 8.7 mg/L RK) with increased proportions of RLAs1642, which was not the case in SM. These results suggest the importance of the media components in the performance of cocultures. Specific initial ratios could either achieve higher HBA titer (6:1 SM, 308.4 mg/L) or RK titer (1:20 SC, 8.7 mg/L) than that of monocultures.

Based on these results, three inoculation ratios (1:1, 1:20, 1:100) were selected for the coculture CL_RK2 in SM (Figure 3a,b). The intermediate *p*-CA was maintained at concentrations (<3.7 mg/L) under different ratios, while HBA levels reduced gradually from 268.2 mg/L at a ratio of 1:1 to 96.9 mg/L at a ratio of 1:100. Similarly, RK titers reduced gradually from 9.7 mg/L at a ratio of 1:1 to 6.6 mg/L at a ratio of 1:100. Although the highest RK titer of coculture CL_RK2 (9.7 mg/L) was lower than that of RLAs1643 monoculture (17.3 mg/L), a great improvement of HBA production was observed, 70% higher than RLAs1643 monoculture (158.8 mg/L) (Figure 3c,e). Interestingly, when comparing the performance of the coculture CL_RK2 in SC and SM (Figure 3d,f), we observed that the levels of *p*-CA and HBA showed a similar trend in different ratios, while those of RK showed an opposite trend and increased gradually from 5.5 mg/L at a ratio of 1:1 to 11.7 mg/L at a ratio of 1:100. The best RK titer of coculture CL_RK2 was found at a 1:100 ratio and was 14% higher than that in the RLAs1643 monoculture (10.3 mg/L). In addition, the coculture showed 5.8 times higher HBA titers than in RLAs1643 monoculture (12.2 mg/L). Interestingly, both the trends of cell growth and RK production at different inoculations differed between SM and SC for the cocultures CL_RK1 and CL_RK2. Although cocultures CL_RK1 and CL_RK2 did not improve the RK titers over monocultures (RLAs1642 and RLAs1643) in SM, HBA accumulated too much higher levels. Moreover, the cocultures CL_RK1 and CL_RK2 demonstrated great potential in producing higher RK titers than in the monocultures from SC.

In addition, we created a three-member coculture, called CL_RK3, which contained a *p*-CA-providing strain RLAs1654 and two RK-producing strains RLAs1642 and RLAs1643. We then tested them at different initial ratios of 1:1:1, 1:20:1, and 10:20:1 (Figure 3a,b). Interestingly, the three-member cocultures appeared to be more robust to variations in the inoculation ratio. In SM, coculture CL_RK3 showed low levels of *p*-CA (< 5 mg/L) but high levels of both RK (13.4 mg/L at 1:20:1) and HBA (280.0 mg/L at 1:20:1) (Figure 3e). Although the maximum RK titer of coculture CL_RK3 was lower than one of the monoculture RLAs1643 (17.3 mg/L), it was higher than the other monoculture RLAs1642 (8.2 mg/L). Overall, CL_RK3 showed increased RK production by 63.8 and 37.5% over the highest titer of CL_RK1 (8.2 mg/L) and CL_RK2 (9.7 mg/L), respectively. CL_RK3 showed similar trends of cell growth, *p*-CA, HBA and RK titers in SC than SM among these three initial ratios. In SC medium, the maximum RK titer of CL_RK3 was 10.0 mg/L, which was slightly lower than that in SM but close to monoculture RLAs1643 in SC (10.3 mg/L). Incidentally, the HBA titer of CL_RK3 in SC was only 29.8 mg/L, 10 times lower than in SM (Figure 3f).

As a summary, the modular coculture strategy demonstrated to be a promising strategy for optimizing metabolic pathways by changing strains, inoculation ratios, and culture media. We found some conditions in which the coculture produced more RK than the monoculture, although this was not the norm, suggesting that division of labor is not always beneficial. Interestingly, cocultures showed generally a much higher titer of HBA (up to 308.4 mg/L) which indicates a better conversion of the sugars into the precursor of RK and suggests that once the current bottleneck (last step in the pathway) is overcome, it can be advantageous to use cocultures.

## Conclusions

In this work, we studied the production of a high-value aromatic, raspberry ketone in the industrial yeast, *S. cerevisiae*, and three different modular approaches were used to optimize production. First, we used modular cloning to generate a combinatorial promoter library that allowed us to optimize the module of RK synthesis. This revealed that the best combination was obtained with low expression of *4CL*, modest expression of *BAS*, and high expression of *RtTAL* and *RiRKS*. Second, a modular pathway strategy enabled the creation of strains with modifications in different metabolic modules. In particular, an RK synthesis module and three additional modules were utilized to increase the production of precursor molecules, including aromatic acids, *p*-CA, and malonyl-CoA. The best engineered strain was that expressing the modules for the synthesis of RK, aromatic acids, and *p*-CA, which achieved the highest reported RK titer of 63.5 mg/L from glucose in *S. cerevisiae*, and the highest yield described in any microorganisms without *p*-CA supplementation (2.1 mg RK/g glucose). Finally, we explored the modularity of cocultures by creating synthetic microbial communities able to produce RK and HBA via division of labor. Variations on culture media, strains used in the coculture, and inoculation ratios influenced production titers of both RK and HBA. Interestingly, some cocultures achieved high HBA titers of up to 308.4 mg/L. This study successfully contributes to the demonstration of the utility of different levels of modularity in synthetic biology tools to optimize metabolic pathways. In addition, these strategies have the potential to be transferred to other pathways and organisms. In particular, three out of the four modules engineered here could be used for the production of a variety of other aromatic compounds of industrial value.

## Materials and Methods

### Strains, Media and Chemicals

*Escherichia coli* Turbo Competent cells (NEB) were used for standard bacterial cloning and plasmid propagation. Selection and growth of *E. coli* was in Lysogeny Broth (LB) medium (VWR) at 37 °C with aeration. Except generating competent cells, the LB medium was supplemented with appropriate antibiotics (ampicillin 100 μg/mL, chloramphenicol 34 μg/mL, or Kanamycin 50 μg/mL).^35^

Model yeast strain BY4741 (MATa his3Δ1 leu2Δ0 met15Δ0 ura3Δ0) was used as the starting wild-type strain in this study, and the list of all engineered yeast strains used in this study are listed in Table S1. Yeast strains were stocked in glycerol to a final concentration of 25% (v/v) at −80 °C. Three culture media were used to maintain yeast cells, including YPD (yeast extract peptone dextrose), SC (synthetic complete dextrose), and SM (synthetic minimal). YPD was made by 10 g/L yeast extract, 20 g/L peptone, and 20 g/L glucose. SC was made by 6.7 g/L yeast nitrogen base without amino acids, 1.4 g/L yeast synthetic drop-out medium supplement without histidine, leucine, tryptophan, and uracil, and 20 g/L glucose. Amino acids and nucleotide were supplemented with histidine (76 mg/L), leucine (380 mg/L), tryptophan (76 mg/L), and uracil (76 mg/L) as the necessary selection markers in yeast transformations. SM was made by 6.7 g/L yeast nitrogen base without amino acids plus 20 g/L glucose. When preparing agar plates, 2% bacteriological agar was added to the above media recipe.

All reagents, chemicals, and analytical standards of 4-hydroxybenzylideneacetone, raspberry ketone, and *p*-coumaric acid are listed as Table S2.

### Plasmid Construction and Bacterial Transformation

All plasmids in this study were created using the MoClo Yeast Toolkit (YTK) system^7^ and the method described in Shaw et al.’s work.^35^ Key gene information in the raspberry ketone synthesis pathway is listed in Table 2 and Table S3; other part or vector sequences in this study can be found either in the YTK system or Shaw’s work.^35^ The full list of all plasmid constructs and oligos used in this study are listed in Tables S4 and S5, respectively. Unless indicated, part sequences were either mutated or synthesized to remove or avoid all instances of the BsmBI, BsaI, BpiI, and NotI recognition sequences.

**Table 2:** Key Gene Information in the Raspberry Ketone Synthesis Pathway (Sequences See Table S3)

module	gene	GenBank accession no.	enzyme	organism	reference
Mod. RK	*RtTAL*	P11544.2	tyrosine ammonia-lyase	*Rhodosporidium toruloides*	(12)
	*At4CL*	Q42524.1	4-coumaroyl-CoA ligase	*Arabidopsis thaliana*	(12)
	*RpBAS*	AAK82824.1	benzalacetone synthase	*Rheum palmatum*	(12)
	*RiRKS*	AEL78826.1	raspberry ketone synthase	*Rubus idaeus*	(12)
Mod. *p*-CA	*FjTAL*	SCV44818.1	tyrosine ammonia-lyase	*Flavobacterium johnsoniae*	(31)
	*VvPAL*	NP_001384847.1	phenylalanine ammonia-lyase	*Vitis vinifera*	(28, 30)
	*AtC4H*	NP_180607.1	cinnamate-4-hydroxylase	*Arabidopsis thaliana*	(13, 29)
Mod. Aro	*ScARO3^K222L^*	NP_010320.3	DAHP synthase	*Saccharomyces cerevisiae*	(23, 27)
	*ScARO4^K229L^*	NP_009808.1	DAHP synthase	*Saccharomyces cerevisiae*	(25, 26)
	*ScARO7^G141S^*	NP_015385.1	chorismate mutase	*Saccharomyces cerevisiae*	(24, 26)
Mod. M-CoA	*ScACC1*^*S659A*,*S1157A*^	CAA96294.1	acetyl-CoA carboxylase	*Saccharomyces cerevisiae*	(33)
	*ScAld6*	NP_015264.1	aldehyde dehydrogenase	*Saccharomyces cerevisiae*	(32)
	*SeACS1^L641p^*	GHM89985.1	acetyl-CoA synthetase	*Salmonella enterica*	(32)

Golden Gate assembly was used to construct all plasmids in Table S4. As described in Shaw’s work,^35^ all parts were set to equimolar concentrations of 50 fmol/mL (50 nM) prior to experiments. Golden Gate reactions were prepared as follows: 0.1 μL of backbone vector, 0.5 μL of each plasmid, 1 μL T4 DNA ligase buffer (Promega), 0.5 μL T7 DNA ligase (NEB), 0.5 μL restriction enzyme (BsaI or BsmBI)(NEB), and water to bring the final volume to 10 μL. Reaction mixtures were then incubated in a thermocycler using the following program: (42 °C for 2 min, 16 °C for 5 min) × 25 cycles followed by a final digestion step of 60 °C for 10 min and then heat inactivation at 80 °C for 10 min. The entire reaction mixture was then ready for *E. coli* transformation, which was followed by a TSS protocol for KCM chemical transformation^36^ before plating on LB plates with the appropriate antibiotics.

### Yeast Transformation and Colony PCR Verification

Yeast transformation was performed by the lithium acetate (LiOAc) protocol.^37^ Chemically competent yeast cells were prepared as follows: fresh isolated colonies were cultured at 30 °C and 250 rpm to saturation overnight in YPD. In the following morning, the cells were diluted 1:100 in 10 mL fresh YPD in a 50 mL conical tube and incubated for 4–6 h to OD 0.8–1.0. Cells were pelleted and washed once with equal volume of 0.1 M LiOAc. Cells were then resuspended in 600 μL of 0.1 M LiOAc, and 100 μL of cells were aliquoted into individual 1.5 mL tubes, pelleted, and ready for yeast transformation. Cells were resuspended in 64 μL of DNA/salmon sperm DNA mixture (10 μL of boiled salmon sperm DNA (Invitrogen) + (NotI digested) plasmids + dd H_2_O) and then mixed with 294 μL of PEG/LiOAc mixture (260 μL 50% (w/v) PEG-3350 + 36 μL 1 M LiOAc). The yeast transformation mixture was then heat-shocked at 42 °C for 40 min, pelleted, resuspended in 200 μL 5 mM CaCl_2_, and waited for 10 min before plating onto the appropriate selection plates. Yeast colonies should come out after the plates were incubated at 30 °C for 2–3 days (or longer for some heavy burden or large genes).

Yeast transformation was verified by colony PCR using the Phire Plant Direct PCR Master Mix (F160L, Thermo Fisher). Three to five isolated colonies for each yeast transformation were selected and resuspended into 20–50 μL of sterile water in PCR tubes. Each 10 μL PCR reaction system included 1 μL of cell suspension, 5 μL of 2× Phire Plant Direct PCR Master Mix, 0.5 μL of forward primer, 0.5 μL of reversed primer, and 3 μL of dd H_2_O. The PCR reactions were performed using the ProFlex PCR System (Thermo Fisher) under the recommended condition of Phire Plant polymerase: initial denaturation at 95 °C and 5 min, followed by 35 cycles of denaturation 98 °C and 5 s, annealing at *X* °C for 5 s, extension at 72 °C and 20 s/kb, and the final extension at 72 °C for 1 min (*X* represents the optimum annealing temperature for each primer pair). The 10 μL PCR reaction was then verified by the agarose gel electrophoresis.

### Yeast Monoculture and Coculture Setup

Fresh isolated colonies of verified engineered yeast strains were precultured in 2 mL of selective SC medium at 30 °C and 250 rpm to saturation overnight. The following morning, 1 mL of preculture was taken and pelleted (5000 rpm, 1 min) in a 1.5 mL tube and then the cell pellet was washed once using SM medium and resuspended again in 1 mL of SM medium. A total of 100 μL of washed cells was diluted 10 times before OD600 nm measurement using cuvettes on a UV/visible spectrophotometer (Biochrom WPA Lightwave II, Biochrom Ltd); the remaining 900 μL of washed cells were then pelleted and resuspended with SM medium to an equivalent OD600 nm value of 10 (OD10) for monoculture and coculture setup.

Both monocultures and cocultures were performed in 96-well deep plates using 500 μL of volume. In monocultures, the initial OD was set as 0.4 for each strain and the 500 μL volume included 20 μL of OD10 individual washed seed culture plus 480 μL of medium (SM or SC). In cocultures, the initial total OD was set as 0.8 (0.4 × 2), and the 500 μL volume included 40 μL of OD10 mixed washed seed cultures plus 460 μL of medium (SM or SC). The strains were inoculated in different initial ratios such as 50:1, 20:1, 6:1, 1:1, 1:20, 1:50, 1:100, etc. The 96-well deep plates were incubated at 30 °C and 250 rpm for 72 h. In addition, the best engineering strain was cultured in a 50 mL flask to verify the RK production titer: 30 °C and 250 rpm in 25 mL of 1.5× synthetic minimal medium (SM) for 72 h.

### OD Measurement

The OD values of seed cultures in tubes were diluted 10 times and measured by a UV/visible spectrophotometer (Biochrom WPA Lightwave II, Biochrom Ltd). The OD values of cultures in 96-well deep plates were measured by a microplate reader (ENZ-INS-A96, ENZO life science). Unless indicated, all reported OD values in the figures were from this microplate reader.

### LC–MS Analysis of Metabolites in the Raspberry Ketone Synthesis Pathway

A total of 300 μL of cell culture was mixed with an equal volume of ethanol by incubating at 700 rpm, 30 °C, and 5 min and then centrifuged at 4000 rpm and 30 min before loading the supernatants into a 96-well sample plate for LC–MS analysis as described earlier.^12^ An Agilent 1290 Infinity system was employed to analyze these prepared samples with an online diode array detector in combination with an Agilent 6500 quadruple time-of-flight (Q-ToF) mass spectrometer. An Agilent Eclipse Plus C18 2.1 × 50 mm (1.8 μm particle size) column was used at a temperature of 25 °C with a solvent flow rate of 0.2 mL/min. LC was performed with a linear gradient of buffer A (0.1% formic acid) and buffer B (0.1% formic acid in acetonitrile) from 2 to 98% buffer B over 2.5 min, which was held at 98% buffer B for 1 min. Injection volume was 1 μL, and spectra were recorded between a mass range of 90 and 1000 *m*/*z* at a rate of 3 spectra per second. The prepared calibration curves of standards include *p*-CA, HBA, and RK. Quantitation was based on the MS peak area of precursor or fragment ion in comparison to the analytical standards. Positive ion detection mode was used for raspberry ketone samples. Error bars represent standard deviation from three independent biological samples.

## Supporting Information Available

(Figure S1) Expression analysis of RK synthesis pathway enzymes; (Figure S2) RK production of strain RLAs1643 in a 50 mL flask; (Table S1) list of all engineered yea st strains used in this study; (Table S2) list of reagents and chemicals; (Table S3) key gene information in the raspberry ketone synthesis pathway; (Table S4) list of all plasmid constructs used in this study; and (Table S5) list of all oligos used in this study (PDF)

## Abbreviations

RK: raspberry ketone
HBA: 4-hydroxy benzalacetone
*p*-CA: *p*-coumaric acid
TAL: tyrosine ammonia lyase
4CL: coumarate-CoA ligase
BAS: benzalacetone synthase
RKS: raspberry ketone reductase
Mod. RK: module of raspberry ketone synthesis
Mod. Aro: module of enhancing aromatic amino acids synthesis
Mod. *p*-CA: module of improving *p*-coumaric acid synthesis
Mod. M-CoA: module of increasing acetyl-CoA and malonyl-CoA supply
*ScARO3^K222L^*,*ScARO4^K229L^*: DAHP synthase from *S. cerevisiae*
*ScARO7^G141S^*: chorismate mutase from *S. cerevisiae*
*VvPAL*: phenylalanine ammonia-lyase from *Vitis vinifera*
*AtC4H*: cinnamate-4-hydroxylase from *Arabidopsis thaliana*
*FjTAL*: tyrosine ammonia-lyase from *Flavobacterium johnsoniae*
*ScALD6*: aldehyde dehydrogenase from *S. cerevisiae*
*SeACS1^L641p^*: acetyl-CoA synthetase with mutate site L641P from *Salmonella enterica*
*ScACC1*^*S659A*,*S1157A*^: acetyl-CoA carboxylase with two mutation sites
S659A: S1157A
