# Supplementary material for: Modular Metabolic Engineering and Synthetic Coculture Strategies for the Production of Aromatic Compounds in Yeast
Source: ACS Synth Biol. 2023 May 23;12(6):1739–49. doi: 10.1021/acssynbio.3c00047 (PMC10278174; doi:10.1021/acssynbio.3c00047)
Supplement: Supplementary file 1 — sb3c00047_si_001.pdf [file sb3c00047_si_001.pdf]

Supporting information for

Modular metabolic engineering and synthetic coculture strategies for the production of aromatic compounds in yeast

Huadong Peng[1, 2, 5 *], Ruiqi Chen[1, 2, 3], William M. Shaw[1, 2], Piotr Hapeta[1, 2], Wei Jiang[1, 2, 5], David J. Bell[1, 4], Tom Ellis[1, 2], Rodrigo Ledesma Amaro[1, 2 *]
[1] Department of Bioengineering, Imperial College London, London SW7 2AZ, UK
[2] Centre for Synthetic Biology, Imperial College London, London SW7 2AZ, UK
[3] College of Life Sciences, Nankai University, Tianjin, 300071, China
[4] SynbiCITE Innovation and Knowledge Centre, Imperial College London, London SW7 2AZ, UK
[5] The Novo Nordisk Foundation Center for Biosustainability, Technical University of Denmark, Kongens Lyngby, 2800, Denmark
* Correspondence: hdpeng89@gmail.com (H.P.), r.ledesma-amaro@imperial.ac.uk (R.L.A.)

Supplementary figure

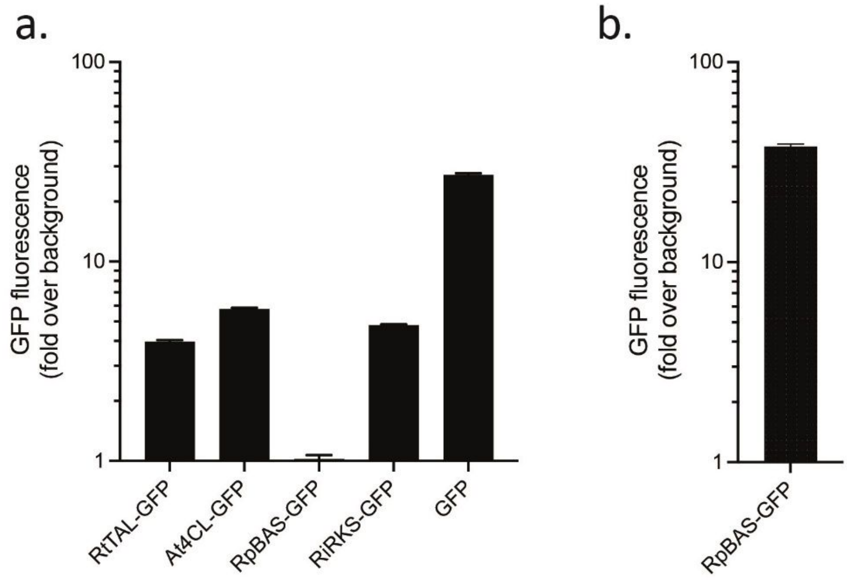

Fig. S1. Expression analysis of RK synthesis pathway enzymes. a. Relative expression of RK synthesis pathway enzymes C-terminally tagged with GFP from Moore et al (2021). **b**. Relative expression of a *S. cerevisiae* codon optimized RpBAS C-terminally tagged with GFP. Experimental measurements are GFP levels per cell determined by flow cytometry and shown as the mean ± SD from six biological replicates.

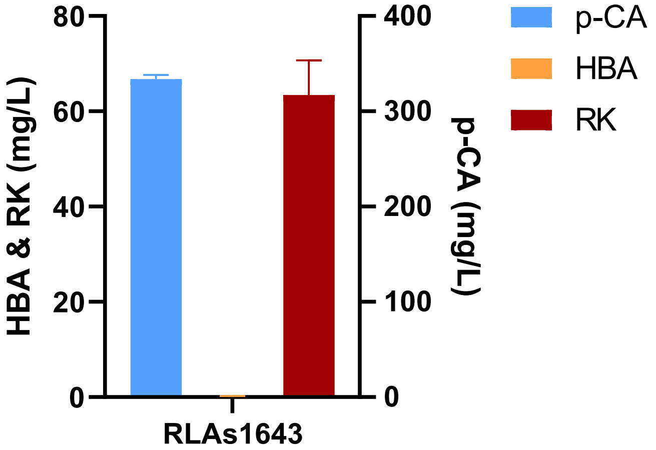

Fig. S2. RK production of strain RLAs1643 in 50 mL flask. Engineering strain RLAs1643 was cultured at 30 °C, 250 rpm in 25 mL 1.5X synthetic minimal medium (SM) in 50 mL flasks for 72 h. The precursors p-coumaric acid (p-CA), 4-hydroxy benzalacetone (HBA) and product RK were shown. These results show the average of 3 replicates and the SD.

Supplementary tables

Table S1 The list of all engineered yeast strains used in this study

Table S2 The list of reagents and chemicals

Table S3 Key gene information in the raspberry ketone synthesis pathway

Table S4 The list of all plasmid constructs used in this study

Table S5 The list of all oligos used in this study

Table S1 The list of all engineered yeast strains used in this study

Strain RLAs No.	Strain No.	Genotype	Yeast marker	Parent strain + Plasmids	Description
20	BY4741△TRP1	*MATa his3Δ1 leu2Δ0 met15Δ0 ura3Δ0ΔTRP1*	-G418	-	Lab stock from yeast knockout library
80	BY4741	*MATa his3Δ1 leu2Δ0 met15Δ0 ura3Δ0*	-	-	Wild type, lab stock
1634	sHP462	*BY4741-pPGK1-RtTAL-tPGK1-pPOP6-At4CL-tADH1-pRNR1-RpBAS-tTDH1-pCCW12-RiRKS-tENO2-vLeu2-pHUM*	-HLUM	BY4741-pHP256-pHUM	↑ Mod. RK: *RtTAL-At4CL-RpBAS-RiRKS*
1640	sHP468	*BY4741△TRP1-pPGK1-RtTAL-tPGK1-pPOP6-At4CL-tADH1-pRNR1-RpBAS-tTDH1-pCCW12-RiRKS-tENO2-vLeu2, pPGK1-FjTAL-tENO1-pCCW12-VvPAL-tSSA-pTDH3-AtC4H-tADH1-vTRP1*	-LT	BY4741△TRP1-pHP256-pHP270	↑ Mod. RK: *RtTAL-At4CL-RpBAS-RiRKS*, Mod.p-CA: *FjTAL-VvPAL-AtC4H*
1641	sHP469	*BY4741△TRP1-pM-pPGK1-RtTAL-tPGK1-pPOP6-At4CL-tADH1-pRNR1-RpBAS-tTDH1-pCCW12-RiRKS-tENO2-vLeu2, pPGK1-FjTAL-tENO1-pCCW12-VvPAL-tSSA-pTDH3-AtC4H-tADH1-vTRP1*	-LTM	BY4741△TRP1-pM-pHP256-pHP270	↑ Mod. RK: *RtTAL-At4CL-RpBAS-RiRKS*, Mod.p-CA: *FjTAL-VvPAL-AtC4H*
1642	sHP470	*sHP468-pHUM*	-HLUMT	sHP468-pHUM	↑ Mod. RK: *RtTAL-At4CL-RpBAS-RiRKS*, Mod.p-CA: *FjTAL-VvPAL-AtC4H*
1643	sHP471	*sHP468-pHM-pCCW12-*$ARO3^{K222L}$*-tENO1-pCCW12-*$ARO4^{K229L}$*-tSSA1-pCCW12-*$ARO7^{G141S}$*-tADH1-vURA3*	-HLUMT	sHP468-pHP125-pHM	↑ Mod. RK: *RtTAL-At4CL-RpBAS-RiRKS*, Mod.p-CA: *FjTAL-VvPAL-AtC4H*, Mod.Aro: $ARO3^{K222L}$-$ARO4^{K229L}$-$ARO7^{G141S}$
1644	sHP472	*sHP469-pCCW12-*$ARO3^{K222L}$*-tENO1- pCCW12-*$ARO4^{K229L}$*-tSSA1-pCCW12-*$ARO7^{G141S}$*-tADH1-vURA3, pTEF1-*$ACC1^{S659A,S1157A}$*-tADH1-vHis3*	-HLUMT	sHP469-pHP125-pHP273	↑ Mod. RK: *RtTAL-At4CL-RpBAS-RiRKS*, Mod.p-CA: *FjTAL-VvPAL-AtC4H*, Mod.Aro: $ARO3^{K222L}$-$ARO4^{K229L}$-$ARO7^{G141S}$, Mod.M-CoA: $ACC1^{S659A,S1157A}$
1645	sHP473	*sHP469-pCCW12-*$ARO3^{K222L}$*-tENO1- pCCW12-*$ARO4^{K229L}$*-tSSA1-pCCW12-*$ARO7^{G141S}$*-tADH1-vURA3,pPGK1-Ald6-tENO1-pTEF1-*$SeACS1^{L641P}$*-tSSA-pTEF1-*$ACC1^{S659A,S1157A}$*-tADH1-vHis3*	-HLUMT	sHP469-pHP125-pHP267	↑ Mod. RK: *RtTAL-At4CL-RpBAS-RiRKS*, Mod.p-CA: *FjTAL-VvPAL-AtC4H*, Mod.Aro: $ARO3^{K222L}$-$ARO4^{K229L}$-$ARO7^{G141S}$, Mod.M-CoA: *ALD6*-$SeACS1^{L641P}$-$ACC1^{S659A,S1157A}$
1646	sHP474	*BY4741-pCCW12-*$ARO3^{K222L}$*-tENO1- pCCW12-*$ARO4^{K229L}$*-tSSA1-pCCW12-*$ARO7^{G141S}$*-tADH1-vURA3-pHM*	-HUM	BY4741-pHP125-pHM	↑ Mod. Aro: $ARO3^{K222L}$-$ARO4^{K229L}$-$ARO7^{G141S}$

1647	sHP475	*BY4741-pPGK1-Ald6-tENO1-pTEF1-SeACS1*^{L641P}*-tSSA-pTEF1-ACC1*^{S659A,S1157A}*-tADH1-vHis3-pUM*	-HUM	BY4741-pHP267-pUM	↑ Mod.M-CoA: *ALD6-SeACS1*^{L641P}*-ACC1*^{S659A,S1157A}
1654	sHP482	*sHP474-pPGK1-FjTAL-tENO1-pCCW12-VvPAL-tSSA-pTDH3-AtC4H-tADH1-vLeu2*	-HLUM	sHP474-pHP271	↑ Mod.p-CA: *FjTAL-VvPAL-AtC4H*
1655	sHP483	*sHP475--pPGK1-RtTAL-tPGK1-pPOP6-At4CL-tADH1-pRNR1-RpBAS-tTDH1-pCCW12-RiRKS-tENO2-vLeu2*	-HLUM	sHP475-pHP256	↑ Mod.M-CoA: *ALD6-SeACS1*^{L641P}*-ACC1*^{S659A,S1157A}, Mod. RK: *RtTAL-At4CL-RpBAS-RiRKS*

Table S2 The list of reagents and chemicals

Reagents and chemicals	Source/supplier	Catalog number
Ampicillin sodium salt	Sigma-Aldrich	A0166-5G
Chloramphenicol	Sigma-Aldrich	C3175-100MG
Kanamycin disulfate salt	Sigma-Aldrich	K1876-5G
Lysogeny Broth (LB)	VWR	J106-500G
Yeast extract	Thermo Fisher	H26769.36
Bacto™ Peptone	Thermo Fisher	211677
Glucose	Thermo Fisher	15023021
Yeast nitrogen base without amino acids	Sigma-Aldrich	Y0626-250g
Yeast synthetic drop-out medium supplement without histidine, leucine, tryptophan and uracil	Sigma-Aldrich	Y2001-20G
L-Histidine	Sigma-Aldrich	H8000-5G
L-Leucine	Sigma-Aldrich	L8912-25G
L-Tryptophan	Sigma-Aldrich	T8941-25G
Uracil	Sigma-Aldrich	U1128-25G
Bacteriological agar	VWR	AAA10752-36
Phire Plant Direct PCR Master Mix	Thermo Fisher	F160L
4-Hydroxybenzylideneacetone, HBA	Thermo Fisher	11408237

Raspberry ketone	Sigma-Aldrich	W258814-25G-K
p-Coumaric acid	Sigma-Aldrich	C9008-5G

Table S3 Key gene information in the raspberry ketone synthesis pathway

Module	Gene name	Enzyme	Organism	Sequence (5' →3')
**Mod. RK**	*RtTAL*	tyrosine ammonia-lyase	*Rhodosporidium toruloides*	ATGGCACCGCGTCCGACCAGCCAGAGCCAGGCACGTACCTGTCCGACCACACAGGTTACCCAGGTTGATATTGTTGAAAAAA<br>TGCTGGCAGCACCGACCGATAGCACCCTGGAACTGGATGGTTATAGCCTGAATCTGGGTGATGTTGTTAGCGCAGCACGTAA<br>AGGTCGTCCGGTTCGTGTTAAAGATAGTGATGAAATTCGCAGCAAAATCGATAAAAGCGTGGAATTTCTGCGTAGCCAGCTG<br>AGCATGAGCGTTTATGGTGTTACCACCGGTTTTGGTGGTAGCGCAGATACCCGTACCGAAGATGCAATTAGCCTGCAGAAAG<br>CACTGCTGGAACATCAGCTGTGTGGTGTTCTGCCGAGCAGCTTTGATAGCTTTCGTCTGGGTCGTGGTCTGGAAAATAGCCTG<br>CCGCTGGAAGTTGTTCGTGGTGCAATGACCATTCGTGTGAATAGCCTGACCCGTGGTCATAGCGCAGTTCGTCTGGTTGTTCT<br>GGAAGCACTGACCAATTTTCTGAATCATGGTATTACCCCGATTGTTCCGCTGCGTGGCACCATTAGCGCAAGCGGTGATCTGA<br>GTCCGCTGAGCTATATTGCAGCAGCAATTAGCGGTCATCCGGATAGCAAAGTTCATGTTGTTCATGAGGGCAAAGAGAAAAT<br>TCTGTACGCACGTGAAGCAATGGCACTGTTTAATCTGGAACCGGTTGTGCTGGGTCCGAAAGAAGGTCTGGGCCTGGTTAAT<br>GGTACAGCAGTTAGCGCCAGCATGGCAACCCTGGCACTGCATGATGCACACATGCTGAGCCTGCTGAGTCAGAGCCTGACCG<br>CAATGACCGTTGAAGCCATGGTTGGTCATGCAGGTAGCTTTCATCCGTTTCTGCACGATGTTACCCGTCCGCATCCGACCCAG<br>ATTGAAGTTGCAGGTAATATTCGTAAACTGCTGGAAGGTAGCCGTTTTGCAGTTCATCATGAAGAAGAGGTGAAAGTCAAAG<br>ATGATGAAGGTATTCTGCGTCAGGATCGTTATCCGCTGCGCACCAGTCCGCAGTGGCTGGGTCCTCTGGTTAGCGATCTGATT<br>CATGCACATGCAGTTCTGACCATTGAAGCAGGTCAGAGTACCACCGATAATCCGCTGATTGATGTTGAAAACAAAACCAGCCA<br>TCATGGTGGTAATTTTCAGGCAGCAGCAGTTGCAAATACCATGGAAAAAACACGCCTGGGTCTGGCACAGATTGGTAAACTG<br>AATTTTACCCAGCTGACCGAAATGCTGAATGCAGGTATGAATCGTGGCCTGCCGAGCTGTCTGGCAGCAGAAGATCCGAGCC<br>TGAGTTATCATTGTAAAGGTCTGGATATTGCCGCAGCCGCATATACCAGCGAACTGGGTCATCTGGCAAATCCGGTTACCACC<br>CATGTTCAGCCTGCCGAAATGGCAAATCAGGCAGTTAATAGCCTGGCCCTGATTAGCGCACGTCGCACCACCGAAAGCAATG<br>ATGTTCTGAGTCTGCTGCTGGCAACCCATCTGTATTGTGTGCTGCAGGCCATTGATCTGCGTGCAATTGAATTTGAGTTCAAAA<br>AACAGTTTGGTCCGGCAATTGTTAGCCTGATTGATCAGCATTTTGGTAGCGCCATGACCGGTAGCAATCTGCGTGATGAGCTG<br>GTTGAAAAAGTGAATAAAACCCTGGCCAAACGTCTGGAACAGACCAATAGCTATGATCTGGTTCCGCGTTGGCATGATGCCT<br>TTAGCTTTGCAGCAGGCACCGTTGTTGAAGTTCTGAGCAGCACCAGCCTGTCACTGGCAGCCGTTAATGCATGGAAAGTTGCA<br>GCGGCAGAAAGCGCAATTAGTCTGACCCGTCAGGTTCGTGAAACCTTTTGGAGCGCAGCAAGCACCAGCAGTCCGGCACTGA<br>GCTATCTGTCACCGCGTACACAGATTCTGTATGCATTTGTTCGTGAAGAACTGGGTGTTAAAGCACGTCGTGGTGATGTTTTTC<br>TGGGTAAACAAGAAGTTACCATTGGTAGCAATGTGAGCAAAATCTATGAAGCCATTAAAAGCGGTCGCATTAATAACGTTCT<br>GCTGAAAATGCTGGCCTAG
	At4CL	4-coumaroyl-CoA ligase	*Arabidopsis thaliana*	ATGGCACCGCAAGAACAGGCAGTTAGCCAGGTTATGGAAAAACAGAGCAACAATAACAACAGCGACGTGATTTTTCGTAGC<br>AAACTGCCGGATATCTATATTCCGAATCATCTGAGCCTGCACGATTATATCTTTCAGAACATTAGCGAGTTTGCCACCAAACCG<br>TGTCTGATTAATGGTCCGACCGGTCATGTTTATACCTATAGTGATGTTCATGTGATCAGCCGTCAGATTGCAGCCAATTTTCAT

			AAACTGGGTGTGAATCAGAATGACGTTGTTATGCTGCTGCTGCCGAATTGTCCGGAATTTGTTCTGAGCTTTCTGGCAGCAAG<br>CTTTCGTGGTGCAACCGCAACCGCAGCAAATCCGTTTTTTACACCGGCAGAAATTGCAAAACAGGCAAAAGCAAGCAACACC<br>AAACTGATTATTACCGAAGCACGTTACGTGGACAAAATCAAACCGCTGCAGAATGATGATGGTGTTGTGATTGTTTGCATCGA<br>TGATAATGAAAGCGTTCCGATTCCGGAAGGTTGTCTGCGTTTTACCGAACTGACCCAGAGCACCACCGAAGCAAGCGAAGTT<br>ATTGATAGCGTTGAAATTAGTCCGGATGATGTTGTTGCACTGCCGTATAGCAGCGGCACCACCGGTCTGCCGAAAGGTGTGA<br>TGCTGACCCATAAAGGTCTGGTTACCAGCGTTGCACAGCAGGTTGATGGTGAAAATCCGAATCTGTATTTTCATAGCGACGAT<br>GTTATTCTGTGTGTGCTGCCGATGTTTCATATTTATGCACTGAATAGCATTATGCTGTGTGGTCTGCGTGTTGGTGCAGCAATT<br>CTGATTATGCCGAAATTTGAAATTAACCTGCTGCTGGAACTGATTCAGCGTTGTAAAGTTACCGTTGCACCGATGGTTCCGCCT<br>ATTGTTCTGGCCATTGCAAAAAGCAGCGAAACCGAAAAATATGATCTGAGCAGCATTCGTGTTGTTAAAAGCGGTGCAGCTC<br>CGCTGGGTAAAGAACTGGAAGATGCAGTTAATGCCAAATTTCCGAATGCAAAACTGGGTCAGGGTTATGGTATGACCGAAG<br>CAGGTCCGGTTCTGGCAATGAGCCTGGGTTTTGCCAAAGAACCGTTTCCGGTTAAATCAGGTGCATGTGGCACCGTTGTTCGT<br>AATGCAGAAATGAAAATCGTTGATCCGGATACCGGTGATAGCCTGAGCCGTAATCAGCCTGGTGAAATTTGTATTCGTGGTC<br>ACCAGATTATGAAAGGCTATCTGAATAATCCGGCAGCCACCGCAGAAACCATTGATAAAGATGGTTGGCTGCATACAGGTGA<br>TATTGGTCTGATTGATGATGATGACGAACTGTTTATTGTGGATCGTCTGAAAGAGCTGATCAAATACAAAGGTTTTCAGGTTG<br>CTCCTGCAGAGCTGGAAGCACTGCTGATTGGTCATCCGGATATTACCGATGTTGCAGTTGTTGCAATGAAAGAAGAAGCAGC<br>AGGCGAAGTTCCGGTTGCATTTGTTGTGAAAAGCAAAGATAGCGAACTGAGCGAAGATGATGTTAAACAGTTTGTTAGCAAA<br>CAGGTGGTGTTCTACAAACGCATCAACAAAGTGTTTTTTACCGAGAGCATTCCGAAAGCACCGAGCGGTAAAATTCTGCGTAA<br>AGACCTGCGTGCCAAACTGGCAAATGGTCTGTAG
RpBAS	benzalacetone synthase	*Rheum palmatum*	ATGGCTACTGAAGAAATGAAGAAATTGGCTACCGTTATGGCTATTGGTACTGCTAATCCACCAAACTGTTATTACCAAGCTGA<br>TTTCCCAGACTTCTACTTCAGAGTTACCAACTCTGATCACTTGATCAACTTGAAGCAAAAGTTCAAGAGATTGTGCGAAAACTC<br>CAGAATCGAAAAGAGATACTTGCACGTTACCGAAGAAATCTTGAAAGAAAACCCAAACATTGCTGCTTACGAAGCTACTTCTT<br>TGAACGTTAGACATAAGATGCAAGTTAAGGGTGTTGCCGAATTGGGTAAAGAAGCTGCTTTGAAAGCAATCAAAGAATGGG<br>GTCAACCTAAGTCCAAGATTACCCATTTGATCGTTTGTTGTTTGGCCGGTGTTGATATGCCAGGTGCTGATTATCAATTGACCA<br>AGTTGTTGGATTTGGACCCATCTGTTAAGAGATTCATGTTCTACCATTTGGGTTGTTATGCTGGTGGTACTGTTTTGAGATTGG<br>CTAAGGATATTGCCGAAAACAACAAGGGTGCTAGAGTTTTGATTGTCTGTTCTGAAATGACTACCACCTGTTTTAGAGGTCCA<br>TCTGAAACTCATTTGGACTCCATGATTGGTCAAGCTATTTTGGGTGATGGTGCTGCTGCTGTTATAGTTGGTGCTGATCCAGAT<br>TTGACTGTTGAAAGACCAATCTTCGAATTGGTTTCTACTGCTCAAACTATCGTTCCAGAATCTCATGGTGCTATTGAAGGTCAT<br>TTGTTGGAATCTGGTTTGTCCTTCCACTTGTACAAAACTGTTCCAACCTTGATCTCCAACAACATTAAGACCTGTTTGTCTGATG<br>CTTTCACCCCATTGAATATCTCTGATTGGAATTCCTTGTTCTGGATTGCTCATCCAGGTGGTCCAGCAATTTTGGATCAAGTTAC<br>TGCTAAAGTTGGTTTGGAAAAAGAAAAGTTGAAGGTTACCAGACAAGTCTTGAAGGATTACGGTAATATGTCCTCTGCTACC<br>GTTTTTTTCATCATGGACGAAATGAGAAAAAAGTCCTTGGAAAATGGTCAAGCCACTACTGGTGAAGGTTTGGAATGGGGTG<br>TTTTGTTTGGTTTTGGTCCAGGTATTACCGTTGAAACTGTTGTCTTGAGATCCGTTCCAGTTATTTCTTAG
RiRKS	raspberry ketone synthase	*Rubus idaeus*	ATGGCAAGCGGTGGTGAAATGCAGGTTAGCAATAAACAGGTGATCTTTCGTGATTATGTTACCGGCTTTCCGAAAGAAAGCG<br>ATATGGAACTGACCACCCGTAGCATTACCCTGAAACTGCCGCAGGGTAGCACCGGTCTGCTGCTGAAAAATCTGTATCTGAGC<br>TGTGATCCGTATATGCGTGCACGTATGACCAATCATCATCGTCTGAGCTATGTGGATAGCTTTAAACCGGGTAGCCCGATTAT<br>TGGTTATGGTGTTGCACGTGTTCTGGAAAGCGGTAATCCGAAATTCAATCCGGGTGATCTGGTTTGGGGTTTTACCGGTTGG

				GAAGAATATAGCGTTATTACCGCAACCGAAAGCCTGTTCAAAATTCATAATACCGATGTTCCGCTGAGCTATTATACAGGTCT<br>GCTGGGTATGCCTGGTATGACCGCCTATGCAGGTTTTTATGAAATTTGCAGCCCGAAAAAAGGCGAAACCGTTTATGTTAGCG<br>CAGCCAGCGGTGCAGTTGGTCAGCTGGTTGGTCAGTTCGCAAAACTGACCGGTTGTTATGTTGTTGGTAGCGCAGGTAGCAA<br>AGAAAAAGTTGATCTGCTGAAAAACAAATTCGGCTTTGATGAGGCCTTCAACTATAAAGAAGAAGCAGATCTGGACGCAGCA<br>CTGCGTCGTTATTTTCCGGATGGTATTGATATCTATTTCGAAAACGTGGGTGGCAAAATGCTGGATGCAGTTCTGCCGAATAT<br>GCGTCCGAAAGGTCGTATTGCAGTTTGTGGTATGATTAGCCAGTATAATCTGGAACAGCCGGAAGGTGTTCGTAATCTGATG<br>GCACTGATTGTTAAACAGGTTCGCATGGAAGGCTTTATGGTCTTTAGCTATTATCACCTGTACGGCAAATTTCTGGAAACCGT<br>GCTGCCGTATATCAAACAGGGTAAAATTACCTATGTGGAAGATGTTGTTGATGGCCTGGATAATGCACCGGCAGCCCTGATT<br>GGTCTGTATAGCGGTCGTAATGTTGGCAAACAGGTTGTTGTTGTTAGCCGTGAATAG
	VvVST1	resveratrol synthase	*Vitis vinifera*	ATGGCCTCTGTGGAAGAGTTCCGAAACGCCCAGCGAGCCAAGGGACCCGCCACCATCCTGGCCATCGGCACCGCTACTCCCG<br>ACCACTGCGTGTACCAGTCTGACTACGCCGACTACTACTTCCGAGTGACCAAGTCTGAGCACATGACCGAGCTGAAGAAGAA<br>GTTCAACCGAATCTGCGACAAGTCTATGATCAAGAAGCGGTACATCCACCTGACCGAGGAAATGCTCGAGGAACACCCCAAC<br>ATCGGCGCCTACATGGCCCCTTCTCTGAACATCCGACAAGAGATCATCACCGCCGAGGTGCCCCGACTGGGCCGAGATGCCG<br>CTCTGAAGGCCCTGAAGGAATGGGGACAGCCCAAGTCCAAGATCACCCACCTGGTGTTCTGCACCACCTCTGGCGTCGAGAT<br>GCCCGGTGCCGACTACAAGCTGGCCAACCTGCTGGGCCTCGAGACTTCTGTGCGACGAGTGATGCTGTACCACCAGGGCTGC<br>TACGCTGGCGGCACCGTGCTGCGAACCGCCAAGGACCTGGCCGAGAACAACGCTGGCGCCCGAGTGCTGGTGGTGTGCTCT<br>GAGATCACCGTGGTGACCTTCCGAGGTCCTTCTGAGGACGCCCTGGACTCTCTGGTCGGACAGGCCCTGTTCGGCGACGGAT<br>CTTCTGCCGTGATCGTGGGCTCTGACCCCGACGTGTCTATCGAGCGACCCCTGTTCCAGCTGGTGTCTGCTGCCCAGACCTTCA<br>TTCCCAACTCTGCCGGCGCTATCGCCGGAAACCTGCGAGAGGTGGGCCTGACCTTCCACCTGTGGCCTAACGTGCCCACTCTG<br>ATCTCTGAGAACATCGAGAAGTGTCTGACCCAGGCTTTCGACCCTCTGGGAATCTCTGACTGGAACTCTCTGTTCTGGATCGC<br>TCACCCCGGTGGACCCGCTATCCTGGACGCCGTCGAGGCCAAGCTGAACCTGGAAAAGAAGAAGCTGGAAGCTACCCGACA<br>CGTGCTGTCTGAGTACGGCAACATGTCCTCTGCCTGCGTGCTGTTCATTCTGGACGAGATGCGAAAGAAGTCTCTGAAGGGC<br>GAGAAGGCCACCACCGGCGAGGGACTCGACTGGGGAGTGCTGTTCGGCTTCGGACCCGGCCTGACCATCGAGACTGTGGTG<br>CTGCACTCTGTGCCCACCGTGACCAACTAG
**Mod. p-CA**	*FjTAL*	tyrosine ammonia-lyase	*Flavobacterium johnsoniae*	ATGAACACCATCAACGAGTACCTGTCTCTGGAAGAGTTCGAGGCCATCATCTTCGGCAACCAGAAGGTGACCATCTCTGACGT<br>GGTGGTGAACCGAGTGAACGAGTCTTTCAACTTCCTGAAGGAATTCTCTGGCAACAAGGTGATCTACGGCGTGAACACCGGC<br>TTCGGCCCCATGGCTCAGTACCGAATCAAGGAATCTGACCAGATCCAGCTGCAGTACAACCTGATCCGATCTCACTCTTCTGG<br>CACCGGCAAGCCTCTGTCTCCCGTGTGCGCCAAGGCCGCCATTCTGGCCCGACTGAACACCCTGTCGCTGGGCAACTCTGGCG<br>TGCACCCCTCTGTGATCAACCTGATGTCTGAGCTGATCAACAAGGACATTACCCCTCTGATCTTCGAGCACGGCGGCGTGGGC<br>GCCTCTGGCGACCTGGTGCAGCTGTCTCACCTGGCTCTGGTGCTGATCGGCGAGGGCGAAGTGTTCTACAAGGGCGAGCGA<br>CGACCCACTCCTGAGGTGTTCGAGATCGAGGGACTGAAGCCCATCCAGGTCGAGATCCGAGAGGGACTCGCCCTGATCAAC<br>GGCACCTCCGTGATGACCGGCATCGGCGTGGTGAACGTGTACCACGCCAAGAAGCTGCTGGACTGGTCCCTGAAGTCCTCTT<br>GCGCCATTAACGAGCTGGTGCAGGCCTACGACGACCACTTCTCTGCCGAGCTGAACCAGACCAAGCGACACAAGGGCCAGC<br>AAGAGATCGCCCTGAAGATGCGACAGAACCTGTCTGACTCTACCCTGATTCGAAAGCGAGAGGACCACCTGTACTCTGGCGA<br>GAACACCGAGGAAATCTTCAAGGAAAAGGTGCAAGAGTACTACTCTCTCCGATGCGTGCCCCAGATTCTGGGCCCCGTGCTG<br>GAAACCATCAACAACGTGGCCTCTATTCTCGAGGACGAGTTCAACTCTGCCAACGACAACCCCATCATCGACGTGAAGAACCA

			GCACGTCTACCACGGCGGCAACTTCCACGGCGACTACATCTCCCTCGAGATGGACAAGCTGAAGATCGTGATCACCAAGCTG<br>ACCATGCTGGCCGAGCGACAGCTGAACTACCTGCTGAACTCTAAGATCAACGAGCTGCTGCCTCCTTTCGTGAACCTGGGCAC<br>CCTGGGCTTCAACTTCGGCATGCAGGGCGTGCAGTTCACCGCCACCTCTACCACCGCCGAGTCTCAGATGCTGTCTAACCCCA<br>TGTACGTGCACTCTATCCCCAACAACAACGATAACCAGGACATCGTGTCTATGGGCACCAACTCCGCCGTGATTACCTCTAAG<br>GTGATCGAGAACGCCTTCGAGGTGCTGGCCATCGAGATGATCACCATCGTGCAGGCCATTGACTACCTGGGCCAGAAGGACA<br>AGATCTCTTCTGTGTCTAAGAAGTGGTACGACGAGATTCGAAACATCATCCCCACCTTTAAGGAAGATCAGGTGATGTACCCC<br>TTCGTGCAGAAGGTCAAGGACCATCTGATTAACAACTAG
VvPAL	phenylalanine ammonia-lyase	*Vitis vinifera*	ATGGACGCCACCAACTGCCACGGTTCCAACAAGGTCGAGTCCTTCTGTGTGTCTGACCCCCTGAACTGGGGCATGGCCGCTG<br>AGACTCTCAAGGGTTCTCACCTGGACGAGGTCAAGCGAATGGTGGCCGAGTACCGAAAGCCCGTCGTCCGACTGGGCGGCG<br>AGACTCTGACCATTTCTCAGGTGGCTGCTATTGCTGGACGAGAGGGCGACGTCGGTGTGGAGCTGTCTGAGACTGCTCGAGC<br>CGGCGTCAACGCTTCCTCTGAGTGGGTCATGGAGTCCATGTCTAAGGGCACCGACTCCTACGGTGTGACCACCGGCTTCGGT<br>GCCACCTCTCACCGACGAACCAAGCAGGGAGGCGCTCTGCAGAAGGAGCTCATTCGATTCCTGAACGCCGGTATCTTCGGAA<br>ACGGACGAGAGTCTTGTCACACCCTGCCTCACTCTGCTACCCGAGCTGCTATGCTCGTCCGAATTAACACCCTGCTCCAGGGA<br>TACTCCGGCATCCGATTCGAGATTCTGGAGGCTATCACCAAGCTGCTCAACCACAACATTACCCCCTGTCTGCCCCTCCGAGGA<br>ACCGTGACCGCTTCTGGCGACCTGGTGCCTCTCTCTTACATCGCCGGTCTGCTCACCGGACGACCCAACTCCAAGGCTGTCGG<br>TCCCTCTGGAGAGGTCGTGAACGCTGAGGAAGCCTTCAAGATGGCCGGCATCGAGTCCGGTTTCTTCGAGCTGCAGCCCAAG<br>GAAGGCCTGGCTCTCGTCAACGGAACCGCCGTGGGTTCCGGACTCGCTTCTATGGTGCTGTTCGAGACTAACGTCCTGGCCG<br>TGCTCTCCGAGGTCCTCTCTGCTATTTTCGCCGAGGTCATGCAGGGCAAGCCCGAGTTCACCGACCACCTCACCCACAAGCTG<br>AAGCACCACCCTGGACAGATTGAGGCCGCTGCCATTATGGAGCACATCCTGGACGGCTCCTCTTACGTCAAGGAAGCCAAGA<br>AGCTCCACGAGATGGACCCCCTGCAGAAGCCCAAGCAGGACCGATACGCTCTCCGAACCTCTCCCCAGTGGCTGGGACCCCA<br>GATCGAGGTCATCCGAGCTTCCACCAAGTCTATTGAGCGAGAGATCAACTCCGTCAACGACAACCCCCTGATTGACGTGTCTC<br>GAAACAAGGCCCTCCACGGTGGAAACTTCCAGGGAACCCCCATCGGTGTCTCCATGGACAACACCCGACTCGCCATCGCTGC<br>CATTGGCAAGCTGATGTTCGCTCAGTTCTCCGAGCTCGTGAACGACTTCTACAACAACGGACTCCCCTCTAACCTGGCTGGCTC<br>CCGAAACCCCTCTCTGGACTACGGCTTCAAGGGTGCTGAGATTGCTATGGCCTCCTACTGCTCTGAGCTGCAGTTCCTCGCCA<br>ACCCCGTCACCAACCACGTGGAGTCCGCTGAGCAGCACAACCAGGACGTCAACTCTCTGGGCCTGATTTCCTCTCGAAAGACC<br>GCTGAGGCCGTCGACATCCTGAAGCTCATGTCCACCACCTACCTGGTGGCTCTCTGTCAGGCCATTGACCTGCGACACCTCGA<br>GGAGAACCTGAAGTCCACCGTCAAGAAAACCGTGTCCCACGTGGCCAAGAAAACCCTGACCATCGGAGCTAACGGCGAGCT<br>CCACCCCTCTCGATTCTGCGAGAAGGACCTGCTCAAGGTCGTGGACCGAGAGCACGTGTTCGCCTACATCGACGACCCCTGTT<br>CCGCTACCTACCCCCTGATGCAGAAGGTGCGACAGGTCCTCGTGGAGCACGCCCTGAACAACGGCGAGTCTGAGAAGAACG<br>GTTCCACCTCTATTTTCCAGAAGATCGGCGCCTTCGAGGAAGAGCTCAAGGCTGTCCTGCCCAAGGAAGTCGAGTCCGCTCGA<br>GATGGCGTCGAGTCCGGAAACCCCTCTATTCCCAACCGAATCAAGGAGTGCCGATCTTACCCCCTGTACAAGTTCGTGCGAGA<br>GGAGCTCGGCACCGGTCTGCTCACCGGAGAGAAGGTCCGATCCCCCGGAGAGGACTTCGACAAGGTGTTCACCGCCATGTG<br>CGAGGGCAAGATCATTGACCCCCTGCTCGACTGTCTGTCTGCTTGGAACGGAGCTCCCCTGCCCATCTGTTAATCTAGTGTCT<br>GTGGTATCTAAGCTATTTATCACTCTTTACAACTTCTACCTCAACTATCTACTTTAATAAATGAATATCGTTTATTCTCTATGATT<br>ACTGTATATGCGTTCCTCTAG

	AtC4H	cinnamate-4-hydroxylase	*Arabidopsis thaliana*	ATGGACCTGCTCCTGCTCGAGAAGTCCCTGATCGCCGTGTTCGTCGCTGTGATTCTGGCCACCGTCATCTCTAAGCTCCGAGG<br>CAAGAAGCTGAAGCTGCCTCCCGGACCCATCCCCATTCCCATCTTCGGAAACTGGCTGCAGGTCGGCGACGACCTGAACCACC<br>GAAACCTCGTGGACTACGCCAAGAAGTTCGGCGACCTCTTCCTGCTCCGAATGGGTCAGCGAAACCTGGTCGTCGTGTCCTCC<br>CCCGACCTCACCAAGGAAGTCCTGCTCACCCAGGGTGTGGAGTTCGGATCCCGAACCCGAAACGTGGTGTTCGACATTTTCAC<br>CGGAAAGGGCCAGGACATGGTGTTCACCGTGTACGGAGAGCACTGGCGAAAGATGCGACGAATCATGACCGTGCCCTTCTT<br>CACCAACAAGGTGGTCCAGCAGAACCGAGAGGGCTGGGAGTTCGAGGCCGCTTCCGTGGTCGAGGACGTCAAGAAGAACC<br>CCGACTCTGCCACCAAGGGTATTGTGCTGCGAAAGCGACTGCAGCTCATGATGTACAACAACATGTTCCGAATCATGTTCGAC<br>CGACGATTCGAGTCCGAGGACGACCCCCTGTTCCTCCGACTGAAGGCTCTGAACGGAGAGCGATCCCGACTCGCCCAGTCTTT<br>CGAGTACAACTACGGAGACTTCATTCCCATCCTGCGACCCTTCCTCCGAGGCTACCTGAAGATTTGCCAGGACGTCAAGGACC<br>GACGAATCGCTCTGTTCAAGAAGTACTTCGTGGACGAGCGAAAGCAGATCGCCTCCTCTAAGCCCACCGGATCTGAGGGCCT<br>GAAGTGTGCTATTGACCACATCCTCGAGGCCGAGCAGAAGGGAGAGATTAACGAGGACAACGTCCTGTACATTGTGGAGAA<br>CATCAACGTCGCCGCTATCGAGACTACCCTGTGGTCCATTGAGTGGGGCATCGCTGAGCTCGTCAACCACCCCGAGATTCAGT<br>CTAAGCTCCGAAACGAGCTGGACACCGTTCTGGGTCCTGGAGTCCAGGTGACCGAGCCTGACCTCCACAAGCTGCCCTACCTC<br>CAGGCTGTGGTCAAGGAGACTCTCCGACTGCGAATGGCCATCCCCCTGCTCGTCCCCCACATGAACCTGCACGACGCCAAGCT<br>CGCTGGCTACGACATTCCCGCCGAGTCCAAGATCCTGGTGAACGCTTGGTGGCTCGCCAACAACCCCAACTCTTGGAAGAAG<br>CCCGAGGAGTTCCGACCCGAGCGATTCTTCGAGGAAGAGTCCCACGTCGAGGCTAACGGTAACGACTTCCGATACGTCCCCT<br>TCGGCGTGGGTCGACGATCTTGCCCCGGAATCATTCTCGCCCTGCCCATTCTGGGCATTACCATCGGTCGAATGGTCCAGAAC<br>TTCGAGCTGCTGCCCCCTCCCGGACAGTCCAAGGTGGACACCTCTGAGAAGGGCGGTCAGTTCTCCCTGCACATCCTCAACCA<br>CTCTATCATTGTGATGAAGCCCCGAAACTGTTAATCTAGTGTCTGTGGTATCTAAGCTATTTATCACTCTTTACAACTTCTACCT<br>CAACTATCTACTTTAATAAATGAATATCGTTTATTCTCTATGATTACTGTATATGCGTTCCTCTAG
**Mod. Aro**	*ScARO3*^{K222L}	DAHP synthase	*Saccharomyces cerevisiae*	ATGTTCATTAAAAACGATCACGCCGGTGACAGGAAACGCTTGGAAGACTGGAGAATCAAAGGTTATGATCCATTAACCCCTC<br>CAGATCTGCTTCAACATGAATTTCCAATTTCAGCCAAAGGTGAGGAAAACATTATCAAGGCAAGAGACTCCGTCTGTGATATT<br>TTGAATGGTAAAGATGATCGTTTAGTTATCGTGATCGGGCCATGTTCCCTACATGACCCCAAAGCCGCTTACGATTACGCTGA<br>CAGATTGGCTAAAATTTCAGAAAAGTTGTCAAAAGACTTATTGATTATTATGAGAGCGTATTTAGAAAAACCAAGGACTACTG<br>TTGGCTGGAAAGGGTTGATTAACGACCCTGATATGAATAACTCTTTTCAAATCAATAAAGGTCTACGGATTTCGAGAGAAATG<br>TTCATAAAACTGGTTGAAAAATTACCCATTGCTGGTGAGATGTTGGATACCATTTCTCCGCAGTTTTTGAGTGATTGTTTCTCCT<br>TGGGTGCCATCGGCGCCAGAACTACTGAATCCCAACTGCACAGAGAATTAGCATCCGGTCTATCTTTCCCTATTGGATTTAAG<br>AACGGTACTGATGGTGGTTTGCAAGTCGCCATCGACGCTATGAGAGCCGCTGCACATGAACATTACTTCCTTTCTGTCACATT<br>GCCAGGTGTCACTGCTATCGTGGGCACTGAAGGTAACAAGGATACCTTCCTGATCTTGAGAGGTGGTAAGAACGGTACTAAC<br>TTTGACAAAGAAAGTGTTCAAAATACTAAGAAACAGTTAGAAAAGGCCGGTTTGACTGATGATTCCCAGAAAAGAATTATGA<br>TCGATTGTTCCCACGGCAACAGTAATAAAGATTTCAAGAACCAACCAAAGGTTGCCAAATGTATTTATGACCAGCTGACGGAG<br>GGTGAGAATAGTCTCTGTGGTGTTATGATTGAGTCCAACATAAATGAAGGTAGACAAGATATTCCCAAAGAAGGTGGCAGA<br>GAGGGATTGAAGTATGGTTGTTCTGTTACGGATGCTTGTATTGGCTGGGAGTCCACCGAACAGGTATTGGAGCTATTGGCAG<br>AAGGTGTTAGAAACAGAAGAAAGGCCTTGAAAAAATAG
	ScARO4^{K229L}	DAHP synthase	*Saccharomyces cerevisiae*	ATGAGTGAATCTCCAATGTTCGCTGCCAACGGCATGCCAAAGGTAAATCAAGGTGCTGAAGAAGATGTCAGAATTTTAGGTT<br>ACGACCCATTAGCTTCTCCAGCTCTCCTTCAAGTGCAAATCCCAGCCACACCAACTTCTTTGGAAACTGCCAAGAGAGGTAGA

				AGAGAAGCTATAGATATTATTACCGGTAAAGACGACAGAGTTCTTGTCATTGTCGGTCCTTGTTCCATCCATGATCTAGAAGC<br>CGCTCAAGAATACGCTTTGAGATTAAAGAAATTGTCAGATGAATTAAAAGGTGATTTATCCATCATTATGAGAGCATACTTGG<br>AGAAGCCAAGAACAACCGTCGGCTGGAAAGGTCTAATTAATGACCCTGATGTTAACAACACTTTCAACATCAACAAGGGTTT<br>GCAATCCGCTAGACAATTGTTTGTCAACTTGACAAATATCGGTTTGCCAATTGGTTCTGAAATGCTTGATACCATTTCTCCTCAA<br>TACTTGGCTGATTTGGTTTCCTTCGGTGCCATTGGTGCCAGAACCACCGAATCTCAACTGCACAGAGAATTGGCCTCCGGTTT<br>GTCTTTCCCAGTTGGTTTCAAGAACGGTACCGATGGTACCTTAAATGTTGCTGTGGATGCTTGTCAAGCCGCTGCTCATTCTCA<br>CCATTTCATGGGTGTTACTTTGCATGGTGTTGCTGCTATCACCACTACTAAGGGTAACGAACACTGCTTCGTTATTCTAAGAGG<br>TGGTAAAAAGGGTACCAACTACGACGCTAAGTCCGTTGCAGAAGCTAAGGCTCAATTGCCTGCCGGTTCCAACGGTCTAATG<br>ATTGACTACTCTCACGGTAACTCCAATAAGGATTTCAGAAACCAACCAAAGGTCAATGACGTTGTTTGTGAGCAAATCGCTAA<br>CGGTGAAAACGCCATTACCGGTGTCATGATTGAATCAAACATCAACGAAGGTAACCAAGGCATCCCAGCCGAAGGTAAAGCC<br>GGCTTGAAATATGGTGTTTCCATCACTGATGCTTGTATAGGTTGGGAAACTACTGAAGACGTCTTGAGGAAATTGGCTGCTGC<br>TGTCAGACAAAGAAGAGAAGTTAACAAGAAATAG
	ScARO7^{G141S}	chorismate mutase	*Saccharomyces cerevisiae*	ATGGATTTCACAAAACCAGAAACTGTTTTAAATCTACAAAATATTAGAGATGAATTAGTTAGAATGGAGGATTCGATCATCTT<br>CAAATTTATTGAGAGGTCGCATTTCGCCACATGTCCTTCAGTTTATGAGGCAAACCATCCAGGTTTAGAAATTCCGAATTTTAA<br>AGGATCTTTCTTGGATTGGGCTCTTTCAAATCTTGAAATTGCGCATTCTCGCATCAGAAGATTCGAATCACCTGATGAAACTCC<br>CTTCTTTCCTGACAAGATTCAGAAATCATTCTTACCGAGCATTAACTACCCACAAATTTTGGCGCCTTATGCCCCAGAAGTTAAT<br>TACAATGATAAAATAAAAAAAGTTTATATTGAAAAGATTATACCATTAATTTCGAAAAGAGATGGTGATGATAAGAATAACTT<br>CTCTTCTGTTGCCACTAGAGATATAGAATGTTTGCAAAGCTTGAGTAGGAGAATCCACTTTGGCAAGTTTGTTGCTGAAGCCA<br>AGTTCCAATCGGATATCCCGCTATACACAAAGCTGATCAAAAGTAAAGATGTCGAGGGGATAATGAAGAATATCACCAATTCT<br>GCCGTTGAAGAAAAGATTCTAGAAAGATTAACTAAGAAGGCTGAAGTCTATGGTGTGGACCCTACCAACGAGTCAGGTGAA<br>AGAAGGATTACTCCAGAATATTTGGTAAAAATTTATAAGGAAATTGTTATACCTATCACTAAGGAAGTTGAGGTGGAATACTT<br>GCTAAGAAGGTTGGAAGAGTAG
**Mod. M-CoA**	*ScACC1*^{S659A,S1157A}	acetyl-CoA carboxylase	*Saccharomyces cerevisiae*	ATGAGCGAAGAAAGCTTATTCGAGTCTTCTCCACAGAAGATGGAGTACGAAATTACAAACTACTCAGAAAGACATACAGAAC<br>TTCCAGGTCATTTCATTGGCCTCAATACAGTAGATAAACTAGAGGAGTCCCCGTTAAGGGACTTTGTTAAGAGTCACGGTGGT<br>CACACGGTCATATCCAAGATCCTGATAGCAAATAATGGTATTGCCGCCGTGAAAGAAATTAGATCCGTCAGAAAATGGGCAT<br>ACGAAACGTTCGGCGATGACAGAACCGTCCAATTCGTCGCCATGGCCACCCCAGAAGATCTGGAGGCCAACGCAGAATATAT<br>CCGTATGGCCGATCAATACATTGAAGTGCCAGGTGGTACTAATAATAACAACTACGCTAACGTAGACTTGATCGTAGACATCG<br>CCGAAAGAGCAGACGTAGACGCCGTATGGGCTGGCTGGGGTCACGCCTCCGAGAATCCACTATTGCCTGAAAAATTGTCCCA<br>GTCTAAGAGGAAAGTCATCTTTATTGGGCCTCCAGGTAACGCCATGAGGTCTTTAGGTGATAAAATCTCCTCTACCATTGTCG<br>CTCAAAGTGCTAAAGTCCCATGTATTCCATGGTCTGGTACCGGTGTTGACACCGTTCACGTGGACGAGAAAACCGGTCTGGTT<br>TCTGTCGACGATGACATCTATCAAAAGGGTTGTTGTACCTCTCCTGAAGATGGTTTACAAAAGGCCAAGCGTATTGGTTTTCCT<br>GTCATGATTAAGGCATCCGAAGGTGGTGGTGGTAAAGGTATCAGACAAGTTGAACGTGAAGAAGATTTCATCGCTTTATACC<br>ACCAGGCAGCCAACGAAATTCCAGGCTCCCCCATTTTCATCATGAAGTTGGCCGGTAGAGCGCGTCACTTGGAAGTTCAACTG<br>CTAGCAGATCAGTACGGTACAAATATTTCCTTGTTCGGTAGAGACTGTTCCGTTCAGAGGCGTCATCAAAAAATTATCGAAGA<br>AGCACCAGTTACAATTGCCAAGGCTGAAACATTTCACGAGATGGAAAAGGCTGCCGTCAGACTGGGGAAACTAGTCGGTTAT<br>GTCTCTGCCGGTACCGTGGAGTATCTATATTCTCATGATGATGGAAAATTCTACTTTTTAGAATTGAACCCAAGATTACAAGTC

GAGCATCCAACAACGGAAATGGTTTCCGGTGTTAACTTACCTGCAGCTCAATTACAAATCGCTATGGGTATCCCTATGCATAG
AATAAGTGACATTAGAACTTTATATGGTATGAATCCTCATTCTGCCTCAGAAATCGATTTCGAATTCAAAACTCAAGATGCCAC
CAAGAAACAAAGAAGACCTATTCCAAAGGGTCATTGTACCGCTTGTCGTATCACATCAGAAGATCCAAACGATGGATTCAAG
CCATCGGGTGGTACTTTGCATGAACTAAACTTCCGTTCTTCCTCTAATGTTTGGGGTTACTTCTCCGTGGGTAACAATGGTAAT
ATTCACTCCTTTTCGGACTCTCAGTTCGGCCATATTTTTGCTTTTGGTGAAAATAGACAAGCTTCCAGGAAACACATGGTTGTT
GCCCTGAAGGAATTGTCCATTAGGGGTGATTTCAGAACTACTGTGGAATACTTGATCAAACTTTTGGAAACTGAAGATTTCGA
GGATAACACTATTACCACCGGTTGGTTGGACGATTTGATTACTCATAAAATGACCGCTGAAAAGCCTGATCCAACTCTTGCCG
TCATTTGCGGTGCCGCTACAAAGGCTTTCTTAGCATCTGAAGAAGCCCGCCACAAGTATATCGAATCCTTACAAAAGGGACAA
GTTCTATCTAAAGACCTACTGCAAACTATGTTCCCTGTAGATTTTATCCATGAGGGTAAAAGATACAAGTTCACCGTAGCTAAA
TCCGGTAATGACCGTTACACATTATTTATCAATGGTTCTAAATGTGATATCATACTGCGTCAACTAGCTGATGGTGGTCTTTTG
ATTGCCATAGGCGGTAAATCGCATACCATCTATTGGAAAGAAGAAGTTGCTGCTACAAGATTATCCGTTGACTCTATGACTAC
TTTGTTGGAAGTTGAAAACGATCCAACCCAGTTGCGTACTCCATCCCCTGGTAAATTGGTTAAATTCTTGGTGGAAAATGGTG
AACACATTATCAAGGGCCAACCATATGCAGAAATTGAAGTTATGAAAATGCAAATGCCTTTGGTTTCTCAAGAAAATGGTATC
GTCCAGTTATTAAAGCAACCTGGTTCTACCATTGTTGCAGGTGATATCATGGCTATTATGACTCTTGACGATCCATCCAAGGTC
AAGCACGCTCTACCATTTGAAGGTATGCTGCCAGATTTTGGTTCTCCAGTTATCGAAGGAACCAAACCTGCCTATAAATTCAA
GTCATTAGTGTCTACTTTGGAAAACATTTTGAAGGGTTATGACAACCAAGTTATTATGAACGCTTCCTTGCAACAATTGATAGA
GGTTTTGAGAAATCCAAAACTGCCTTACTCAGAATGGAAACTACACATCTCTGCTTTACATTCAAGATTGCCTGCTAAGCTAGA
TGAACAAATGGAAGAGTTAGTTGCACGTTCTTTGAGGCGTGGTGCTGTTTTCCCAGCTAGACAATTAAGTAAATTGATTGATA
TGGCCGTGAAGAATCCTGAATACAACCCCGACAAATTGCTGGGCGCCGTCGTGGAACCATTGGCGGATATTGCTCATAAGTA
CTCTAACGGGTTAGAAGCCCATGAACATTCTATATTTGTCCATTTCTTGGAAGAATATTACGAAGTTGAAAAGTTATTCAATGG
TCCAAATGTTCGTGAGGAAAATATCATTCTGAAATTGCGTGATGAAAACCCTAAAGATCTAGATAAAGTTGCGCTAACTGTTT
TGTCTCATTCGAAAGTTTCAGCGAAGAATAACCTGATCCTAGCTATCTTGAAACATTATCAACCATTGTGCAAGTTATCTTCTA
AAGTTTCTGCCATTTTCTCTACTCCTCTACAACATATTGTTGAACTAGAATCTAAGGCTACCGCTAAGGTCGCTCTACAAGCAA
GAGAAATTTTGATTCAAGGCGCTTTACCTTCGGTCAAGGAAAGAACTGAACAAATTGAACATATCTTAAAATCCTCTGTTGTG
AAGGTTGCCTATGGCTCATCCAATCCAAAGCGCTCTGAACCAGATTTGAATATCTTGAAGGACTTGATCGATTCTAATTACGTT
GTGTTCGATGTTTTACTTCAATTCCTAACCCATCAAGACCCAGTTGTGACTGCTGCAGCTGCTCAAGTCTATATTCGTCGTGCTT
ATCGTGCTTACACCATAGGAGATATTAGAGTTCACGAAGGTGTCACAGTTCCAATTGTTGAATGGAAATTCCAACTACCTTCA
GCTGCGTTCTCCACCTTTCCAACTGTTAAATCTAAAATGGGTATGAACAGGGCTGTTGCTGTTTCAGATTTGTCATATGTTGCA
AACAGTCAGTCATCTCCGTTAAGAGAAGGTATTTTGATGGCTGTGGATCATTTAGATGATGTTGATGAAATTTTGTCACAAAG
TTTGGAAGTTATTCCTCGTCACCAATCTTCTTCTAACGGACCTGCTCCTGATCGTTCTGGTAGCTCCGCATCGTTGAGTAATGTT
GCTAATGTTTGTGTTGCTTCTACAGAAGGTTTCGAATCTGAAGAGGAAATTTTGGTAAGGTTGAGAGAAATTTTGGATTTGAA
TAAGCAGGAATTAATCAATGCTTCTATCCGTCGTATCACATTTATGTTCGGTTTTAAAGATGGGTCTTATCCAAAGTATTATACT
TTTAACGGTCCAAATTATAACGAAAATGAAACAATTCGTCACATTGAGCCGGCTTTGGCCTTCCAACTGGAATTAGGAAGATT
GTCCAACTTCAACATTAAACCAATTTTCACTGATAATAGAAACATCCATGTCTACGAAGCTGTTAGTAAGACTTCTCCATTGGA
TAAGAGATTCTTTACAAGAGGTATTATTAGAACGGGTCATATCCGTGATGACATTTCTATTCAAGAATATCTGACTTCTGAAGC
TAACAGATTGATGAGTGATATATTGGATAATTTAGAAGTCACCGACACTTCAAATTCTGATTTGAATCATATCTTCATCAACTT

			CATTGCGGTGTTTGATATCTCTCCAGAAGATGTCGAAGCCGCCTTCGGTGGTTTCTTAGAAAGATTTGGTAAGAGATTGTTGA<br>GATTGCGTGTTTCTTCTGCCGAAATTAGAATCATCATCAAAGATCCTCAAACAGGTGCCCCAGTACCATTGCGTGCCTTGATCA<br>ATAACGTTTCTGGTTATGTTATCAAAACAGAAATGTACACCGAAGTCAAGAACGCAAAAGGTGAATGGGTATTTAAGTCTTTG<br>GGTAAACCTGGATCCATGCATTTAAGACCTATTGCTACTCCTTACCCTGTTAAGGAATGGTTGCAACCAAAACGTTATAAGGC<br>ACACTTGATGGGTACCACATATGTCTATGACTTCCCAGAATTATTCCGCCAAGCATCGTCATCCCAATGGAAAAATTTCTCTGC<br>AGATGTTAAGTTAACAGATGATTTCTTTATTTCCAACGAGTTGATTGAAGATGAAAACGGCGAATTAACTGAGGTGGAAAGA<br>GAACCTGGTGCCAACGCTATTGGTATGGTTGCCTTTAAGATTACTGTAAAGACTCCTGAATATCCAAGAGGCCGTCAATTTGT<br>TGTTGTTGCTAACGATATCACATTCAAGATCGGTTCCTTTGGTCCACAAGAAGACGAATTCTTCAATAAGGTTACTGAATATGC<br>TAGAAAGCGTGGTATCCCAAGAATTTACTTGGCTGCAAACTCAGGTGCCAGAATTGGTATGGCTGAAGAGATTGTTCCACTAT<br>TTCAAGTTGCATGGAATGATGCTGCCAATCCGGACAAGGGCTTCCAATACTTATACTTAACAAGTGAAGGTATGGAAACTTTA<br>AAGAAATTTGACAAAGAAAATTCTGTTCTCACTGAACGTACTGTTATAAACGGTGAAGAAAGATTTGTCATCAAGACAATTAT<br>TGGTTCTGAAGATGGGTTAGGTGTCGAATGTCTACGTGGATCTGGTTTAATTGCTGGTGCAACGTCAAGGGCTTACCACGAT<br>ATCTTCACTATCACCTTAGTCACTTGTAGATCCGTCGGTATCGGTGCTTATTTGGTTCGTTTGGGTCAAAGAGCTATTCAGGTC<br>GAAGGCCAGCCAATTATTTTAACTGGTGCTCCTGCAATCAACAAAATGCTGGGTAGAGAAGTTTATACTTCTAACTTACAATT<br>GGGTGGTACTCAAATCATGTATAACAACGGTGTTTCACATTTGACTGCTGTTGACGATTTAGCTGGTGTAGAGAAGATTGTTG<br>AATGGATGTCTTATGTTCCAGCCAAGCGTAATATGCCAGTTCCTATCTTGGAAACTAAAGACACATGGGATAGACCAGTTGAT<br>TTCACTCCAACTAATGATGAAACTTACGATGTAAGATGGATGATTGAAGGTCGTGAGACTGAAAGTGGATTTGAATATGGTTT<br>GTTTGATAAAGGGTCTTTCTTTGAAACTTTGTCAGGATGGGCCAAAGGTGTTGTCGTTGGTAGAGCCCGTCTTGGTGGTATTC<br>CACTGGGTGTTATTGGTGTTGAAACAAGAACTGTCGAGAACTTGATTCCTGCTGATCCAGCTAATCCAAATAGTGCTGAAACA<br>TTAATTCAAGAACCTGGTCAAGTTTGGCATCCAAACTCCGCCTTCAAGACTGCTCAAGCTATCAATGACTTTAACAACGGTGAA<br>CAATTGCCAATGATGATTTTGGCCAACTGGAGAGGTTTCTCTGGTGGTCAACGTGATATGTTCAACGAAGTCTTGAAGTATGG<br>TTCGTTTATTGTTGACGCATTGGTGGATTACAAACAACCAATTATTATCTATATCCCACCTACCGGTGAACTAAGAGGTGGTTC<br>ATGGGTTGTTGTCGATCCAACTATCAACGCTGACCAAATGGAAATGTATGCCGACGTCAACGCTAGAGCTGGTGTTTTGGAAC<br>CACAAGGTATGGTTGGTATCAAGTTCCGTAGAGAAAAATTGCTGGACACCATGAACAGATTGGATGACAAGTACAGAGAATT<br>GAGATCTCAATTATCCAACAAGAGTTTGGCTCCAGAAGTACATCAGCAAATATCCAAGCAATTAGCTGATCGTGAGAGAGAA<br>CTATTGCCAATTTACGGACAAATCAGTCTTCAATTTGCTGATTTGCACGATAGGTCTTCACGTATGGTGGCCAAGGGTGTTATT<br>TCTAAGGAACTGGAATGGACCGAGGCACGTCGTTTCTTCTTCTGGAGATTGAGAAGAAGATTGAACGAAGAATATTTGATTA<br>AAAGGTTGAGCCATCAGGTAGGCGAAGCATCAAGATTAGAAAAGATCGCAAGAATTAGATCGTGGTACCCTGCTTCAGTGG<br>ACCATGAAGATGATAGGCAAGTCGCAACATGGATTGAAGAAAACTACAAAACTTTGGACGATAAACTAAAGGGTTTGAAATT<br>AGAGTCATTCGCTCAAGACTTAGCTAAAAAGATCAGAAGCGACCATGACAATGCTATTGATGGATTATCTGAAGTTATCAAGA<br>TGTTATCTACCGATGATAAAGAAAAATTGTTGAAGACTTTGAAATAG
ScAld6	aldehyde dehydrogenase	*Saccharomyces cerevisiae*	ATGTCTTCTGCCACCACTGAAGATGTTGAATATGCTATCGAATGTGCCGACCGTGCTTTCCACGACACTGAATGGGCTACCCA<br>AGACCCAAGAGAAAGAGGCCGTCTACTAAGTAAGTTGGCTGACGAATTGGAAAGCCAAATTGACTTGGTTTCTTCCATTGAA<br>GCTTTGGACAATGGTAAAACTTTGGCCTTAGCCCGTGGGGATGTTACCATTGCAATCAACTGTCTAAGAGATGCTGCTGCCTA<br>TGCCGACAAAGTCAACGGTAGAACAATCAACACCGGTGACGGCTACATGAACTTCACCACCTTAGAGCCAATCGGTGTCTGT<br>GGTCAAATTATTCCATGGAACTTTCCAATAATGATGTTGGCTTGGAAGATCGCCCCAGCATTGGCCATGGGTAACGTCTGTAT

			CTTGAAACCCGCTGCTGTCACACCTTTAAATGCCCTATACTTTGCTTCTTTATGTAAGAAGGTTGGTATTCCAGCTGGTGTCGTC<br>AACATCGTTCCAGGTCCTGGTAGAACTGTTGGTGCTGCTTTGACCAACGACCCAAGAATCAGAAAGCTGGCTTTTACCGGTTC<br>TACAGAAGTCGGTAAGAGTGTTGCTGTCGACTCTTCTGAATCTAACTTGAAGAAAATCACTTTGGAACTAGGTGGTAAGTCCG<br>CCCATTTGGTCTTTGACGATGCTAACATTAAGAAGACTTTACCAAATCTAGTAAACGGTATTTTCAAGAACGCTGGTCAAATTT<br>GTTCCTCTGGTTCTAGAATTTACGTTCAAGAAGGTATTTACGACGAACTATTGGCTGCTTTCAAGGCTTACTTGGAAACCGAAA<br>TCAAAGTTGGTAATCCATTTGACAAGGCTAACTTCCAAGGTGCTATCACTAACCGTCAACAATTCGACACAATTATGAACTACA<br>TCGATATCGGTAAGAAAGAAGGCGCCAAGATCTTAACTGGTGGCGAAAAAGTTGGTGACAAGGGTTACTTCATCAGACCAAC<br>CGTTTTCTACGATGTTAATGAAGACATGAGAATTGTTAAGGAAGAAATTTTTGGACCAGTTGTCACTGTCGCAAAGTTCAAGA<br>CTTTAGAAGAAGGTGTCGAAATGGCTAACAGCTCTGAATTCGGTCTAGGTTCTGGTATCGAAACAGAATCTTTGAGCACAGG<br>TTTGAAGGTGGCCAAGATGTTGAAGGCCGGTACCGTCTGGATCAACACATACAACGATTTTGACTCCAGAGTTCCATTCGGTG<br>GTGTTAAGCAATCTGGTTACGGTAGAGAAATGGGTGAAGAAGTCTACCATGCATACACTGAAGTAAAAGCTGTCAGAATTAA<br>GTTGTAG
SeACS1^{L641p}	acetyl-CoA synthetase	*Salmonella enterica*	ATGTCACAAACACACAAACATGCTATTCCTGCGAATATCGCTGACAGGTGCTTAATCAACCCTGAACAATACGAAACGAAGTA<br>CAAGCAGTCTATCAACGATCCTGATACTTTCTGGGGCGAGCAAGGTAAGATACTCGATTGGATTACTCCATATCAAAAGGTCA<br>AAAACACATCCTTTGCTCCTGGAAATGTGTCAATCAAGTGGTACGAGGACGGCACTCTAAACCTAGCTGCTAATTGCTTGGAT<br>CGACACCTCCAGGAAAATGGTGACAGAACGGCAATCATTTGGGAAGGTGATGATACTTCTCAATCTAAGCACATCTCCTACA<br>GAGAGTTACACAGAGATGTTTGCAGATTCGCGAATACTTTACTGGACCTGGGTATCAAAAAGGGCGATGTTGTGGCAATCTA<br>CATGCCTATGGTCCCAGAGGCAGCTGTGGCAATGTTGGCCTGTGCCAGAATAGGAGCAGTCCATAGCGTTATCTTTGGCGGA<br>TTCTCCCCTGAAGCCGTTGCTGGGAGAATCATTGACTCATCAAGTAGATTAGTTATCACTGCCGACGAAGGTGTTAGAGCAGG<br>TAGATCCATCCCATTGAAGAAAAACGTTGATGACGCGTTGAAAAACCCAAACGTTACGAGTGTGGAGCATGTAATTGTACTA<br>AAGCGTACCGGCTCTGATATAGACTGGCAGGAAGGTAGGGATTTGTGGTGGAGAGATCTTATTGAGAAAGCAAGTCCAGAA<br>CACCAACCAGAAGCAATGAATGCGGAAGATCCATTGTTCATCTTGTATACATCTGGGTCAACTGGCAAACCAAAAGGTGTTTT<br>GCATACAACAGGTGGTTATCTCGTATACGCCGCAACAACCTTTAAGTACGTTTTTGATTACCATCCAGGTGATATCTACTGGTG<br>TACCGCTGATGTCGGTTGGGTTACTGGTCATAGTTACCTGCTTTACGGTCCACTGGCATGCGGCGCAACCACTTTGATGTTTG<br>AAGGAGTACCAAACTGGCCAACCCCAGCCAGGATGTGTCAAGTGGTCGATAAACACCAAGTGAACATATTGTACACAGCCCC<br>AACCGCCATTAGAGCGCTAATGGCCGAAGGAGATAAGGCGATTGAGGGAACAGATAGAAGTAGCCTACGTATCTTAGGATC<br>CGTTGGCGAGCCAATCAATCCAGAAGCTTGGGAATGGTATTGGAAAAAGATTGGTAAGGAAAAGTGTCCAGTAGTGGATAC<br>ATGGTGGCAAACTGAAACAGGTGGATTCATGATTACACCTCTTCCAGGTGCAATAGAATTGAAGGCTGGGTCTGCTACTAGG<br>CCTTTCTTCGGCGTCCAACCTGCTTTAGTAGACAACGAAGGGCATCCACAAGAGGGGGCAACAGAAGGCAATCTAGTGATAA<br>CTGATTCCTGGCCTGGTCAGGCTAGAACATTGTTTGGTGATCACGAAAGATTCGAACAAACCTATTTCTCAACTTTCAAAAACA<br>TGTATTTCAGCGGTGACGGTGCGAGAAGAGATGAAGATGGGTACTACTGGATTACCGGCAGAGTAGATGACGTCCTTAACG<br>TATCTGGACATCGTCTGGGTACAGCTGAGATTGAGTCAGCTTTAGTTGCTCATCCTAAGATTGCTGAAGCTGCAGTCGTTGGC<br>ATCCCACACGCTATCAAGGGTCAAGCCATATACGCATATGTTACACTCAACCATGGTGAGGAACCATCTCCAGAGCTATACGC<br>AGAGGTCAGAAATTGGGTTCGAAAGGAAATAGGGCCTTTAGCCACACCAGATGTTTTGCATTGGACAGATTCATTGCCTAAG<br>ACAAGATCTGGAAAGATTATGAGGCGTATACTTAGAAAGATCGCCGCCGGAGATACGTCTAACTTAGGTGATACTTCTACTCT<br>TGCCGATCCAGGCGTGGTCGAAAAACCTTTAGAGGAAAAACAAGCTATTGCTATGCCATCATAG

Table S4 The list of all plasmid constructs used in this study

Plasmid RLAp No.	Plasmid No.	Description	Parts information						Vector		Antibiotic marker
			Part 2		Part 3		Part 4				
433	pM	Addgene #64179 (Mülleder et al. 2016)									*AmpR*
423	pHM	Addgene #64173 (Mülleder et al. 2016)									*AmpR*
434	pUM	Addgene #64176 (Mülleder et al. 2016)									*AmpR*
428	pHUM	Addgene #64170 (Mülleder et al. 2016)									*AmpR*
1615	pHP120	*pCCW12-ARO3* ^{K222L}*-tENO1- S/1 [cas]*	pYTK010	*pCCW12*	pHP059	*ARO3*^{K222L}	pYTK051	*tENO1*	pWS041	pWS041 - S/1 [cas]	*AmpR*
1618	pHP123	*pCCW12-ARO4*^{K229L}*-tSSA1- 1/2 [cas]*	pYTK010	*pCCW12*	pHP060	*ARO4*^{K229L}	pYTK052	*tSSA1*	pWS043	pWS043 - 1/2 [cas]	*AmpR*
1619	pHP124	*pCCW12-ARO7*^{G141S}*-tADH1- 2/E [cas]*	pYTK010	*pCCW12*	pHP061	*ARO7*^{G141S}	pYTK053	*tADH1*	pWS044	pWS044 - 2/E [cas]	*AmpR*
1620	pHP125	*pCCW12-ARO3* ^{K222L}*-tENO1- pCCW12-ARO4*^{K229L}*-tSSA1-pCCW12-ARO7*^{G141S}*-tADH1-vURA3*	pHP120 + pHP123 + pHP124						pYTK096	URA3 Integratio n vector	*KanR*
1702	pHP235	*FjTAL-pYTK001*									*CamR*
1705	pHP238	*VvPAL-pYTK001*									*CamR*
1706	pHP239	*AtC4H-pYTK001*									*CamR*
1711	pHP244	*pPGK1-FjTAL-tENO1-S/1 [cas]*	pYTK011	*pPGK1*	pHP235	*FjTAL*	pYTK051	*tENO1*	pWS041	WS041 - S/1 [cas]	*AmpR*
1715	pHP248	*pSM785-RtTAL [3]*									*CamR*
1716	pHP249	*pSM786-At4CL [3]*									*CamR*
1717	pHP250	*pWS537-RpBAS [3]*									*CamR*
1718	pHP251	*PSM788-RiRKS [3]*									*CamR*
1719	pHP252	*pPGK1-RtTAL-tPGK1-S/1 [cas]*	pYTK011	*pPGK1*	pHP248	*RtTAL*	pYTK054	*tPGK1*	pWS041	WS041 - S/1 [cas]	*AmpR*
1720	pHP253	*pPOP6-At4CL-tADH1-1/2 [cas]*	pYTK024	*pPOP6*	pHP249	*At4CL*	pYTK053	*tADH1*	pWS043	pWS043 - 1/2 [cas]	*AmpR*
1721	pHP254	*pRNR1-RpBAS-tTDH1- 2/3 [cas]*	pYTK021	*pRNR1*	pHP250	*RpBAS*	pYTK056	*tTDH1*	pWS045	pWS045 – 2/3 [cas]	*AmpR*

1722	pHP255	*pCCW12-RiRKS-tENO2-3/E [cas]*	pYTK010	*pCCW12*	pHP251	*RiRKS*	pYTK055	*tENO2*	pWS046	pWS046 – 3/E [cas]	*AmpR*
1723	pHP256	*pPGK1-RtTAL-tPGK1-pPOP6-At4CL-tADH1-pRNR1-RpBAS-tTDH1-pCCW12-RiRKS-tENO2-vLEU2*	pHP252 + pHP253+ pHP254 + pHP255						pWS064	LEU2 Integratio n Vector	*KanR*
1728	pHP261	*Ald6-pYTK001*	pIYC05 as template, Addgene #64742 (Chen et al 2013)						pYTK001	entry vector	*CamR*
1729	pHP262	*SeACS-pYTK001*	pIYC05 as template, Addgene #64742 (Chen et al 2013)						pYTK001	entry vector	*CamR*
1730	pHP263	*ACC1*^{S659A,S1157A}*-pYTK001*	pAD as template, Addgene #64747 (Shi et al 2014)						pYTK001	entry vector	*CamR*
1731	pHP264	*pPGK1-Ald6-tENO1 -S/1 [cas]*	pYTK011	*pPGK1*	pHP261	*Ald6*	pYTK051	*tENO1*	pWS041	WS041 - S/1 [cas]	*AmpR*
1732	pHP265	*pTEF1-SeACS1*^{L641P}*-tSSA- 1/2 [cas]*	pYTK013	*pTEF1*	pHP262	*SeACS*^{L641P}	pYTK052	*tSSA1*	pWS043	pWS043 - 1/2 [cas]	*AmpR*
1733	pHP266	*pTEF1-ACC1*^{S659A,S1157A}*-tADH1-2/E [cas]*	pYTK013	*pTEF1*	pHP263	*ACC1*^{S659A,S1157A}	pYTK053	*tADH1*	pWS044	pWS044 - 2/E [cas]	*AmpR*
1734	pHP267	*Ald6-SeACS1-ACC1-vHis3*	pHP264 + pHP265 + pHP266						pWS065	HIS3 Integratio n vector	*KanR*
1735	pHP268	*pCCW12-VvPAL-tSSA- 1/2 [cas]*	pYTK010	*pCCW12*	pHP238	*VvPAL-pYTK001*	pYTK052	*tSSA1*	pWS043	pWS043 - 1/2 [cas]	*AmpR*
1736	pHP269	*pTDH3-AtC4H-tADH1-2/E [cas]*	pYTK009	*pTDH3*	pHP239	*AtC4H-pYTK001*	pYTK053	*tADH1*	pWS044	pWS044 - 2/E [cas]	*AmpR*
1737	pHP270	*pPGK1-FjTAL-tENO1-pCCW12-VvPAL-tSSA-pTDH3-AtC4H-tADH1-vTRP1*	pHP244 + pHP268 + pHP269						pWS1290	TRP1 integratio n vector	*KanR*
1738	pHP271	*pPGK1-FjTAL-tENO1-pCCW12-VvPAL-tSSA-pTDH3-AtC4H-tADH1-vLEU2*	pHP244 + pHP268 + pHP269						pWS064	LEU2 Integratio n Vector	*KanR*
1739	pHP272	*pCCW12-ARO3* ^{K222L}*-tENO1- pCCW12-ARO4*^{K229L}*-tSSA1-pCCW12-ARO7*^{G141S}*-tADH1-vHIS3*	pHP120 + pHP123 + pHP124						pWS065	HIS3 Integratio n vector	*KanR*
1740	pHP273	*pTEF1-ACC1*^{S659A,S1157A}*-tADH1-vHIS3*	pYTK013	*pTEF1*	pHP263	*ACC1*^{S659A,S1157A}	pYTK053	*tADH1*	pWS065	HIS3 Integratio n vector	*KanR*

1905	pHP274	*pPGK1-RtTAL-tPGK1-pPOP6-At4CL-tADH1-pRNR1-RpBAS-tTDH1-pCCW12-RiRKS-tENO2-vURA3*	pHP252 + pHP253+ pHP254 + pHP255	pWS064	LEU2 Integratio n Vector	*KanR*

Table S5 The list of all oligos used in this study

Oligos RLA ID.	Primer No. & Description	Sequence (5' →3')	Tm °C	Notes
966	oHP005 URA3 5’ forward	GGGCGGATTACTACCGTT	60.67°C	
967	oHP006 URA3 5’ reverse	GTAATGTTATCCATGTGGGC	58.53°C	
968	oHP007 URA3 3’ forward	AGAGCACTTGAATCCACTGC	61.35°C	
969	oHP008 URA3 3’ reverse	GATTTGGTTAGATTAGATATGGTTTC	58.39°C	
970	oHP009 LEU2 5’ forward	CATAAATACCTTTCAAGC	49.02°C	
971	oHP010 LEU2 5’ reverse	TACAATCCTTGCCCGTGATG	64.95°C	Verify the integration sites of URA3, LEU2 and HO
972	oHP011 LEU2 3’ forward	ACTCGTATCGCATGTCGGTG	65.29°C	
973	oHP012 LEU2 3’ reverse	CTTCTTATGTTTTACATG	44.17°C	
974	oHP013 HO 5’ forward	CACATCATTTTCGTGGATCC	62.06°C	
975	oHP014 HO 5’ reverse	ACAGCGATGGAACTTACGGC	65.48°C	
976	oHP015 HO 3’ forward	TATCGTGTTGCATCTGCGGC	68.52°C	
977	oHP016 HO 3’ reverse	CTTTGGACTTAAAATGGCGT	59.93°C	
1483	oHP516 RtTAL Fwd	GCAATGATGTTCTGAGTCTG	59°C	
1484	oHP517 Rt4CL Rev	GTAACGTGCTTCGGTAATAATC	59.6°C	
1485	oHP518 Rt4CL Fwd	GTTAAAAGCGGTGCAGCTC	62.9°C	Verify pHP256 Raspberry Ketone synthesis pathway
1486	oHP519 BAS Rev	GATTGCTTTCAAAGCAGCTTC	63.3°C	
1487	oHP520 BAS Fwd	GAATCTCATGGTGCTATTGAAGG	63.9°C	
1488	oHP521 RKS Rev	GTTGAAGGCCTCATCAAAGC	64.1°C	
1496	oHP529 Ald6 Fwd1	TGGTCTCGTCTCGtcgggcatGGTCTCATATGACTAAGCTACACTTTGACACTG	55.9°C	*Ald6* gene PCR amplification from template pIYC05, Addgene #64742
1497	oHP530 Ald6 Rev1	TGGTCTCGTCTCGGAAACCTCACAAACGGTGTT	58.5°C	
1498	oHP531 Ald6 Fwd2	TGGTCTCGTCTCGTTTCTTCTGCCACCACTGAA	61.2°C	
1499	oHP532 Ald6 Rev2	TGGTCTCGTCTCGggtcatgcGGTCTCAGGATCCCAACTTAATTCTGACAGCTTTT	57.4°C	
1500	oHP533 SeACSL641P Fwd1	ACTTCCCGTCTCGtcgggcatGGTCTCATATGTCACAAACACACAAACATG	56.8°C	*SeACS*^{L641P} gene PCR amplification from template pIYC05,
1501	oHP534 SeACSL641P Rev1	ACTTCCCGTCTCGTCATCTCTTCTCGCACCGTC	61.1°C	
1502	oHP535 SeACSL641P Fwd2	ACTTCCCGTCTCCATGAAGATGGGTACTACTGG	52.2°C	

1503	oHP536 SeACSL641P Rev2	ACTTCCCGTCTCCGCCTCATAATCTTTCCAGA	52.9°C	Addgene #64742
1504	oHP537 SeACSL641P Fwd3	ACTTCCCGTCTCGAGGCGTATACTTAGAAAGATCGC	54.0°C	
1505	oHP538 SeACSL641P Rev3	ACTTCCCGTCTCGggtcatgcGGTCTCAGGATCCTGATGGCATAGCAATAGC	57.9°C	
1506	oHP539 ACC1S659A,S1157A Fwd1	CTGTCTCGTCTCGtcgggcatGGTCTCATATGagcgaagaaagcttattc	54.4°C	
1507	oHP540 ACC1S659A,S1157A Rev1	CTGTCTCGTCTCGaacgtTTCgtatgcccattttct	58.6°C	
1508	oHP541 ACC1S659A,S1157A Fwd2	CTGTCTCGTCTCGcgttcggcgatgacagaa	66.7°C	
1509	oHP542 ACC1S659A,S1157A Rev2	CTGTCTCGTCTCGacagaAACcagaccggttttctcg	64.4°C	
1510	oHP543 ACC1S659A,S1157A Fwd3	CTGTCTCGTCTCCctgtcgacgatgacatct	57.7°C	
1511	oHP544 ACC1S659A,S1157A Rev3	CTGTCTCGTCTCCgCCTctgaacggaacagtc	58.9°C	
1512	oHP545 ACC1S659A,S1157A Fwd4	CTGTCTCGTCTCGAGGcgtcatcaaaaaattatcga	58.2°C	
1513	oHP546 ACC1S659A,S1157A Rev4	CTGTCTCGTCTCGgaAACcatttccgttgttgg	61.3°C	*ACC1*^{S659A,S1157A} gene PCR amplification from template pAD, Addgene #64747
1514	oHP547 ACC1S659A,S1157A Fwd5	CTGTCTCGTCTCGTTtccggtgttaacttacct	55.1°C	
1515	oHP548 ACC1S659A,S1157A Rev5	CTGTCTCGTCTCGCTcaaagaacgtgcaact	57.0°C	
1516	oHP549 ACC1S659A,S1157A Fwd6	CTGTCTCGTCTCCtgAGGcgtggtgctgttttccca	66.4°C	
1517	oHP550 ACC1S659A,S1157A Rev6	CTGTCTCGTCTCCggtcatgcGGTCTCAGGATCCtttcaaagtcttcaacaatttttctt	62.3°C	
1519	oHP552 ACC1S659A,S1157A Rev3	TACGGACGTCTCCggtcCCTctgaacggaacagtc	58.9°C	
1520	oHP553 ACC1S659A,S1157A Fwd4	GTGATGCGTCTCGtcggcgtcatcaaaaaattatcga	58.2°C	
1522	oHP555 ACC1S659A,S1157A verif Rev1	gatagcttgagcagtcttgaag	60.3C	
1523	oHP556 ACC1S659A,S1157A verif Rev2	cagtacgttcagtgagaacag	58.12°C	
1524	oHP557 ACC1S659A,S1157A verif Rev3	gcaatgaagttgatgaagatatg	60.49°C	
1525	oHP558 ACC1S659A,S1157A verif Rev4	gtccttcaagatattcaaatctgg	61.96°C	
1526	oHP559 SeACSL641P Fwd4	GTACTAAAGCGTACCGGC	58°C	
1527	oHP560 Ald6 Fwd3	CACTAACCGTCAACAATTCG	61.1°C	
1528	oHP561 SeACSL641P Rev4	GAGAATCCGCCAAAGATAAC	60.6°C	Verify pHP267
1529	oHP562 SeACSL641P Fwd5	CGGCAGAGTAGATGACGTC	61.6°C	
1530	oHP563 ACC1** Rev7	gcgtctacgtctgctctttc	62.2°C	
1531	oHP564 PAL rev1	GACTCCATGACCCACTCAG	61.3°C	Verify pHP270, pHP271
1532	oHP565 C4H rev 1	GTACACGGTGAACACCATGTC	63.3°C	
1533	oHP566 ARO3 Fwd	GTATTTATGACCAGCTGACGG	61.4°C	Verify pHP125
1534	oHP567 ARO4 Rev	CTAGATCATGGATGGAACAAG	59.1°C	

1535	oHP568 ARO4 Fwd	GTTTGTGAGCAAATCGCTAAC	61.7°C
1536	oHP569 ARO7 Rev	CTTCTGATGCGAGAATGC	60.2°C

References

M. Mülleder, K. Campbell, O. Matsarskaia, F. Eckerstorfer and M. Ralser, F1000Research, 2016, 5.
Y. Chen, L. Daviet, M. Schalk, V. Siewers and J. Nielsen, Metabolic Engineering, 2013, 15, 48-54.
S. Shi, Y. Chen, V. Siewers and J. Nielsen, mBio, 2014, 5, e01130-01114.
